# Eleven genes associated with progression and prognosis of endometrial cancer (EC) identified by comprehensive bioinformatics analysis

**DOI:** 10.1186/s12935-019-0859-1

**Published:** 2019-05-20

**Authors:** JinHui Liu, ShuLin Zhou, SiYue Li, Yi Jiang, YiCong Wan, XiaoLing Ma, WenJun Cheng

**Affiliations:** Department of Gynecology, The First Affiliated Hospital of Nanjing Medical University, Nanjing, 210029 Jiangsu China

**Keywords:** Bioinformatics analysis, Endometrial cancer, PPI, WGCNA

## Abstract

**Background:**

Endometrial cancer (EC) is one of the female malignant tumors. Endometrial cancer predominately affects post-menopausal women. Bioinformatics analysis has been widely applied to screen and analyze genes in linkage to various types of cancer progression.

**Methods:**

Download the gene expression profile from Gene Expression Omnibus (GEO). Calculate raw expression data according to pre-processing procedures. We performed the “limma” R language package to screen DEGs between Endometrial cancer tissue samples and normal uterus tissue samples. Enrichment of the functions and pathways was analyzed by using clusterprofiler. We utilized Search Tool for the Retrieval of Interacting Genes Database (STRING) to assess protein–protein interaction (PPI) information, and then we used plug-in Molecular Complex Detection (MCODE) to screen hub modules of PPI network in Cytoscape. We also performed functional analysis on the genes in the hub module by using clusterprofiler. Next, we utilized the “WGCNA” package in R to establish co-expression network for the DEGs. The Venn diagram was performed to overlap the gene in key module and hub PPI cluster. We validated the key genes in TCGA, GEPIA, UALCAN and Immunohistochemistry staining obtained from The Human Protein Atlas database. And then we did ROC curve analysis by SPSS. Gene set enrichment analysis (GSEA) and mutation analysis were also performed for hub genes.

**Results:**

Functional and pathway enrichment analysis demonstrated that the upregulated differentially expressed genes (DEGs) were significantly enriched in CXCR chemokine receptor binding, chemokine activity, chemokine receptor binding, G-protein coupled receptor binding, RAGE receptor binding, cytokine activity, microtubule binding, receptor regulator activity and microtubule motor activity, and the down-regulated genes were highly enriched in collagen binding. After using STRING software to construct PPI network, 30 prominent proteins were identified and the first two significant modules were selected. In co-expression network, 5 EC-related modules were identified. Among them, the turquoise module has the highest correlation with the EC. We further analyzed the genes in the PPI and turquoise module, and selected eleven key genes related to EC after validation of TCGA database, GEPIA, UALCAN and immunohistochemistry. Six of them had mutation significance.

**Conclusions:**

In summary, these 11 genes may become new therapy targets for EC treatment.

**Electronic supplementary material:**

The online version of this article (10.1186/s12935-019-0859-1) contains supplementary material, which is available to authorized users.

## Background

Endometrial cancer is the most common malignancy of the female genital tract [1]. Endometrial cancer predominately affects post-menopausal women, however 15–25% of cases are diagnosed before menopause. Endometrial cancer is not amenable to screening, hence effective management is required once diagnosed.

Due to the limitation of experiment, Bioinformatics Analysis has been widely applied to screen and analyze genes in linkage to various types of cancer progression [2, 3]. For example, Omer et al. released that the homeostasis of cholesterol greatly contributed to the development of cancers by using TCGA database [4]. Many genes with similar expression patterns actually affect each other and even have a regulatory relationship [5]. Most studies only focus on the differential expression of genes, but ignore the chain between genes. Weighted gene expression network analysis (WGCNA) is a systematic biological method which is utilized to describe the pattern of gene association between different samples [6]. It can be used to identify highly synergistically altered gene sets and candidate biomarker genes or therapeutic targets based on the associations of gene sets and associations between gene sets and phenotypes. Recently WGCNA is comprehensively used in cancer-related research [7]. For instance, Zhou et al. revealed that TOP2A might be used as a potential biological target for the prognosis and progression of pancreatic ductal adenocarcinoma [8]. Wang et al. found that ASPM may cause cirrhosis, then further produce hepatocellular carcinoma [9]. In this research, we first screened differentially expressed genes by using WGCNA-based systems biology methods, and then constructed PPI network and co-expression network of genes. Ultimately we discovered the essential genes and pathways involved in the carcinogenic mechanism of EC [10].

## Methods

### Data collection

We downloaded the gene expression profile from the Gene Expression Omnibus (GEO) database (https://www.ncbi.nlm.nih.gov/geo/). Dataset GSE17025 was processed by Affymetrix Human Gene 2.0 ST Array [transcript (gene) version] (Affymetrix, Santa Clara, CA, USA). In order to identify hub genes and pathways in this research, we used the processed data to filter DEGs, set up PPI networks and co-expression networks. Dataset GSE17025 includes 91 tumor tissue samples and 12 normal tissue samples.

### Research design and data preprocessing

**Fig. 1:**
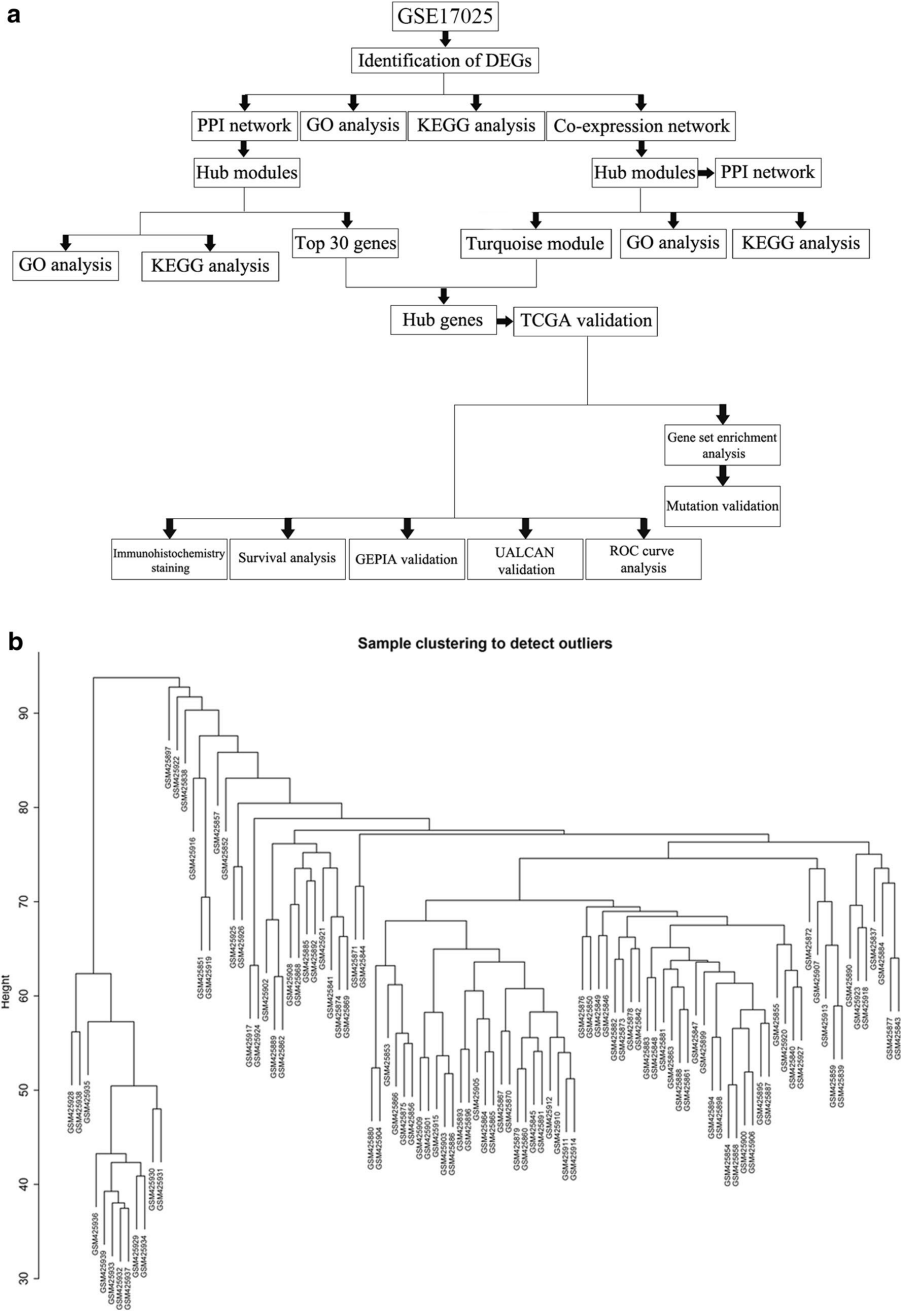
Study design and data preprocessing. **a** Flow diagram of study. **b** Samples clustering of GSE17025 to detect outliers

The research was designed according to the flow chart (Fig. 1a). Pre-processing procedures was used to process raw data, including RMA background correction, and the “affy” R language package also was applied to complete log2 transformation, quantile normalization and median polish algorithm summarization. Probes were annotated by the Affymetrix annotation files. Microarray quality was evaluated by sample clustering in light of the distance between different samples in Pearson’s correlation matrices. We did not delete any of the samples from the subsequent analysis in the test dataset (Fig. 1b).

### Differentially expressed genes (DEGs)

We utilized the “limma” R language package to screen the DEGs between endometrial cancer samples and normal uterus samples. The adjust *p* < 0.05 and |log2fold change (FC)| > 1 were chosen as the cut-off criteria.

### GO term and KEGG pathway enrichment analysis

To further explore the biological significance of DEGs, we used a package called clusterprofiler which has the ability to analyze and visualize data for enrichment analysis of functions and pathways. In GO and KEGG analysis, we obtained more valuable information. A *p*-value < 0.05 was considered a significant enrichment.

### Comprehensive analysis of PPI network

We used Search Tool for the Retrieval of Interacting Genes Database (STRING) (https://www.string-db.org/) to assess protein–protein interaction (PPI) information [11]. In addition, in order to explore the relationship between DEGs, we used the STRING database and converted the results visually by using Cytoscape software. Confidence score > 0.7 was set as significant [12].

### Hub module selection and functional analysis

Plug-in Molecular Complex Detection (MCODE) was utilized to choose hub modules of PPI network in Cytoscape with a degree cut-off = 2, node score cut-off = 0.2, k-core = 2, and max. Depth = 100 as the criterion [13]. Next we used the clusterprofiler to perform functional analysis of the genes in the hub module.

### Co-expression network creation and module functional analysis

The first step was to test the expression data profile of the DEGs in order to see whether they were suitable samples and genes. Next, we used the "WGCNA" package in the R language to create the co-expression network for DEGs [14, 15]. The Pearson’s correlation matrices were both functioned for all pair-wise genes. After that, a power function amn = |cmn|β (cmn = Pearson’s correlation between gene m and gene n; amn = adjacency between gene m and gene n) was utilized to erect a weighted adjacency matrix. We used a soft-thresholding parameter β to emphasize strong correlations between genes and penalize weak correlations. Then, we converted the adjacency to topological overlap matrix (TOM) to measure network connectivity of a gene which was defined as the sum of its adjacency with all other genes for network generation. We created average linkage hierarchical clustering due to the TOM-based dissimilarity measure with a minimum size (gene group) of 50 for the genes dendrogram, thereby classifying genes with similar expression profiles into the same gene module. And then we calculated the dissimilarity of module eigengenes. In order to find relevant modules that have an impact on EC, we further conducted functional enrichment analysis on these gene modules.

### Validation of hub genes

The key genes were identified as the intersecting genes of the turquoise module in WGCNA and PPI hub genes. These data were come from the TCGA databases, and validation was performed by R package. The Venn diagram was drawn through an online website (http://bioinfogp.cnb.csic.es/tools/venny/index.html) [16] to overlap the gene in hub module and PPI hub cluster. The hub gene was finally validated in GEPIA (Gene Expression Profiling Interactive Analysis) [17] and UALCAN (http://ualcan.path.uab.edu/analysis.html) [18]. The Human Protein Atlas (HPA) (https://www.proteinatlas.org/) were used to validate the expression of the real hub genes [19]. ROC curve was plotted to evaluate the capability of distinguishing tumor and normal tissues.

### Gene set enrichment analysis (GSEA)

In validation set GSE17025, samples of EC were divided into two groups respectively according to the expression level of the real hub genes. To identify potential function of the hub genes, GSEA (https://software.broadinstitute.org/gsea/index.jsp) was conducted to detect whether a series of priori defined biological processes were enriched in the gene rank derived from DEGs between the two groups [20]. Terms enriched in all real hub genes with FDR < 0.05 were identified.

### Statistical analysis

All analysis were conducted three times and represented data from three separate experiments. Two-tailed Student’s t-test was utilized for significance of differences between subgroups. Statistical analysis processed via SPSS 16.0. Statistical significance was set at probability values of *p* < 0.05.

## Results

### Identification of DEGs in EC and the enrichment of these genes

We analyzed the DEGs of GSE17025 by using the limma package. We used *p* < 0.05 and |logFC| ≥ 1 as the cutoff criteria. We screened 1,737 DEGs, including 690 up-regulated genes and 1048 down-regulated genes in EC samples compared with normal uterus samples (Additional file 1: Fig. S1A). We identified all the DEGs which were shown in the above volcano map according to the value of |logFC| and then displayed the top 200 DEGs on a heatmap (Additional file 1: Fig. S1B). The clusterprofiler package was applied to compare gene clusters according to their enriched biological processes, with a cutoff criteria of *p* < 0.05. In GO analysis, the upregulated genes were mostly enriched in CXCR chemokine receptor binding, chemokine activity, chemokine receptor binding, G-protein coupled receptor binding, RAGE receptor binding, cytokine activity, microtubule binding, receptor regulator activity and microtubule motor activity (Additional file 1: Fig. S2A), and the down-regulated genes were highly enriched in collagen binding (Additional file 1: Fig. S2B). In the KEGG analysis, the upregulated genes were mostly enriched in IL-17 signaling pathway and Chemokine signaling pathway (Additional file 1: Fig. S2C). The down-regulated genes were mostly enriched in MAPK signaling pathway and TGF-beta signaling pathway (Additional file 1: Fig. S2D). The above enrichment analysis can help us further study the role of DEGs in EC.

### PPI network and cluster analysis

**Fig. 2:**
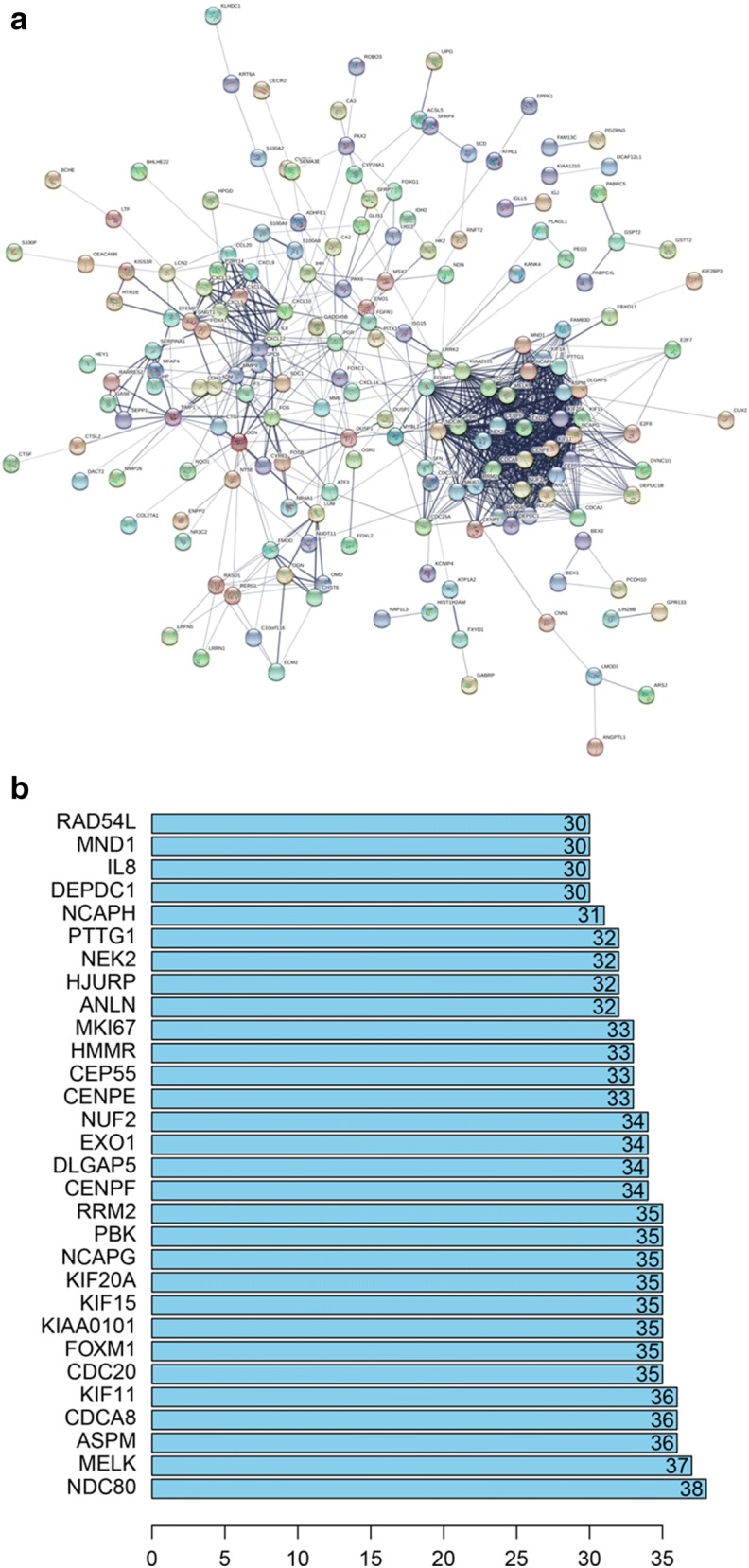
Cluster analysis of the PPI network. **a** 1737 DEGs were filtered into the DEGs PPI network complex that contained 256 nodes and 877 edges. **b** Histogram of key proteins. The y-axis represents the name of genes, the x-axis represent the number of adjacent genes, and height is the number of gene connections

**Fig. 3:**
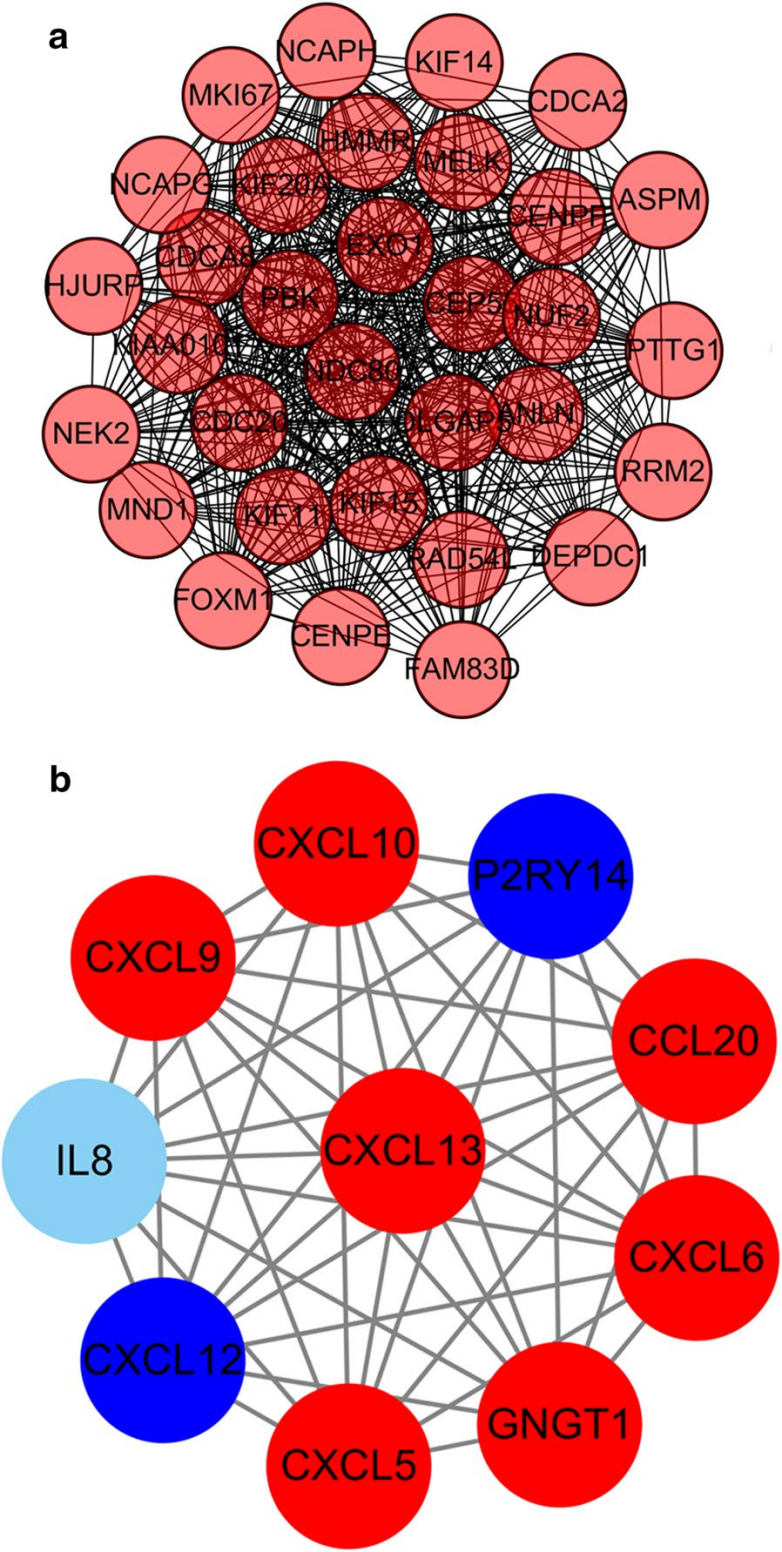
Module analysis of PPI network. The red node represents the up-regulated gene and the blue node represents the down-regulated gene. **a** Module rank 1. This cluster consists of 32 nodes and 477 edges and has the highest score in those clusters. **b** Module rank 2

Via the STRING website, 1737 DEGs were screened into the DEGs PPI network complex, which contained 256 nodes and 877 edges (Fig. 2a). After that, we applied the MCODE, a plug-in used to score and find parameters that had been optimized to produce the best results for the network, to find clusters in the network. Eight clusters were calculated according to k-core = 2. Among them, cluster 1, including 32 nodes and 477 edges, got the highest score in these clusters (Fig. 3a). Cluster 2 including 10 nodes and 45 edges, got the highest score in these clusters (Fig. 3b). This result may suggest that the above 42 DEGs played a critical role in EC.

After using STRING software to perform PPI analysis, 30 prominent proteins were identified. In these identified proteins, estrogen NDC80 and MELK were relatively important. NDC80 was considered to be the most important protein and contacted 38 nodes (Fig. 2b).

### Hub module selection and validation

Degree cut-off = 2, node score cut-off = 0.2, k-core = 2, and max. Depth = 100 were set as the criterion. Top 2 significant modules were selected by using plug-in MCODE. GO analysis and KEGG analysis of each module were performed by clusterprofiler (Additional file 1: Figs. S3, S4).

### Weighted co-expression network construction and analysis

**Fig. 4:**
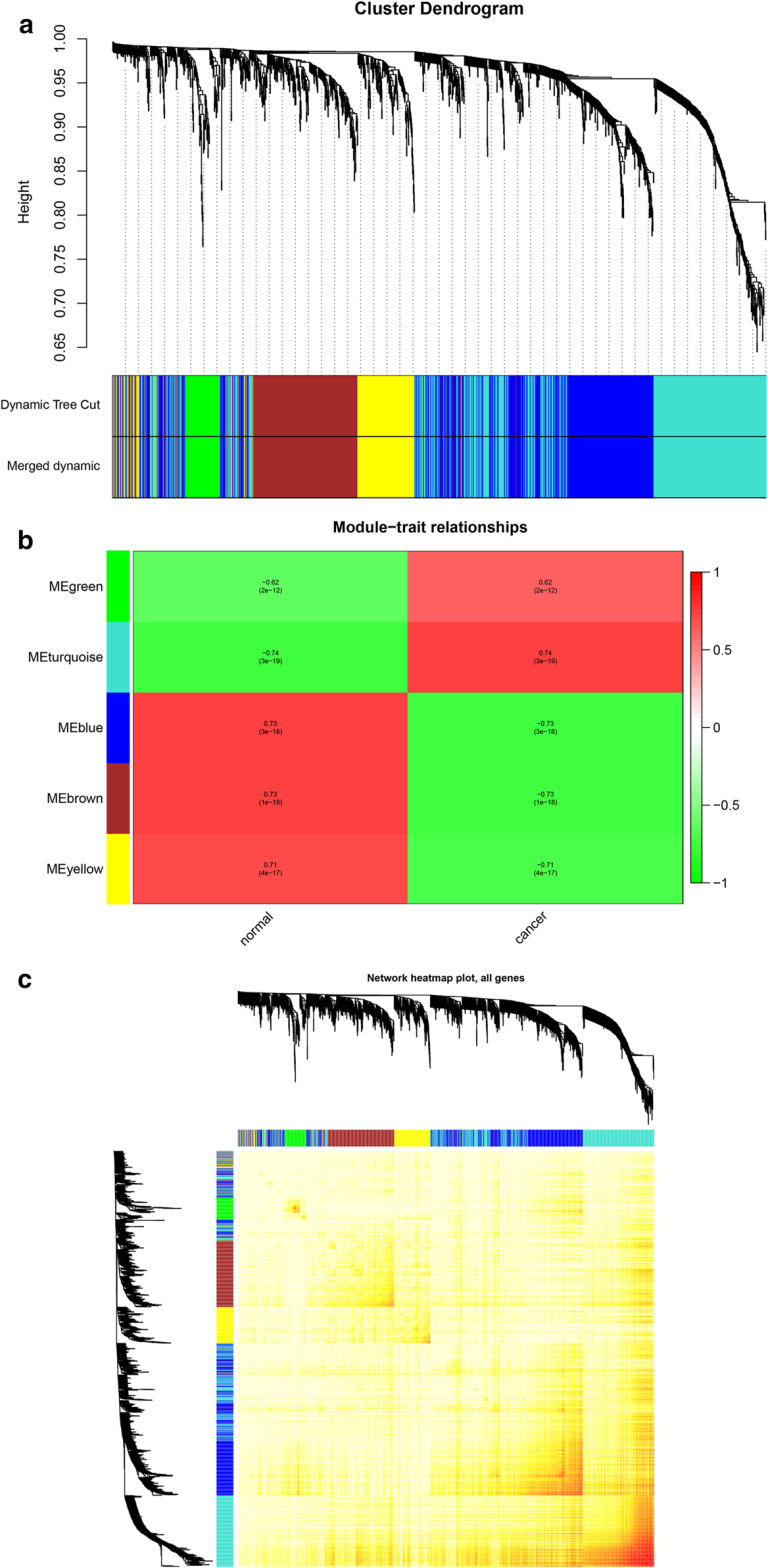
Hub module selection. **a** Dendrogram of all differentially expressed genes clustered based on a dissimilarity measure (1-TOM). **b** Correlation between modules and traits. The upper number in each cell refers to the correlation coefficient of each module in the trait, and the lower number is the corresponding *p*-value. Among them, the turquoise module was the most relevant modules with cancer traits. **c** A heatmap of all genes. The intensity of the red color indicates the strength of the correlation between pairs of modules on a linear scale

We first evaluated the quality for the expression data matrix of GSE17025, and then conducted the "WGCNA" package in the R language. Also, we selected the power β = 4 (scale free R^2 = 0.89) to ensure a scalefree network (Also, we selected the power Fig. S5A–D). After removing the batch effect, we preprocessed the data and then further analyzed the modules with highly related genes, 5 modules were excavated (Fig. 4a). Among the modules, module turquoise has the highest correlation with cancer traits (Fig. 4b). All genes were identified for the heatmap (Fig. 4c).

**Fig. 5:**
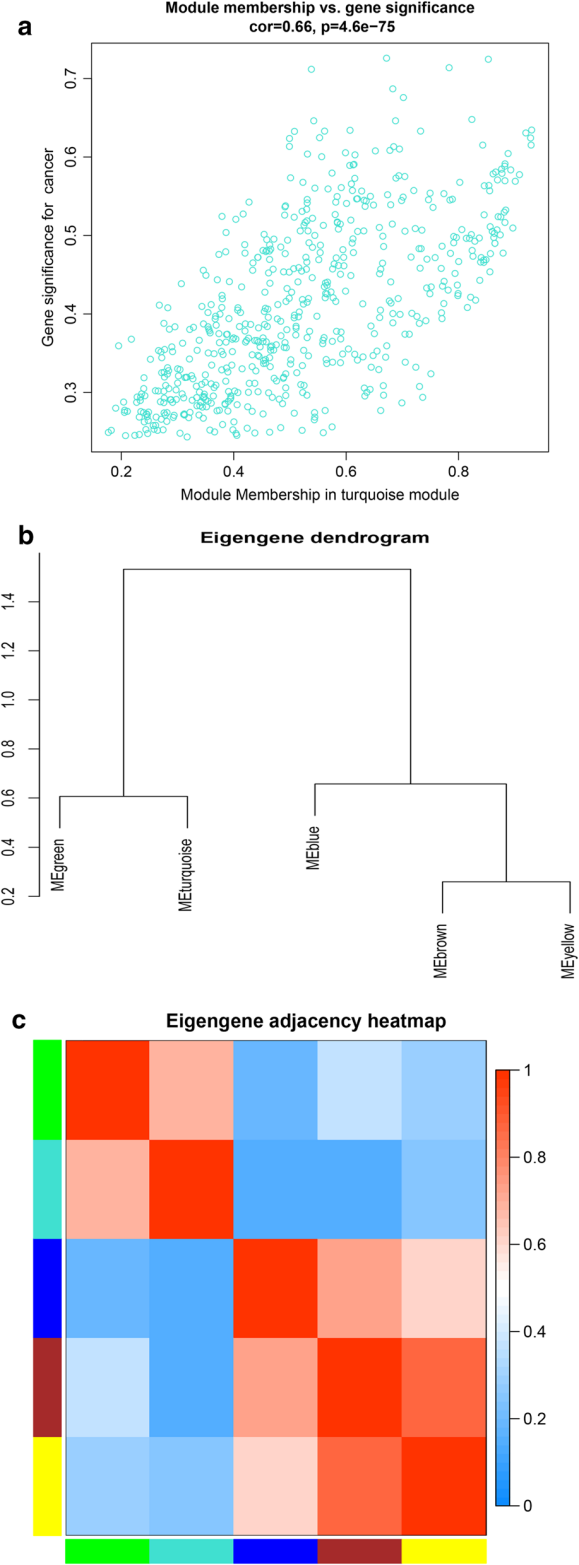
Select hub genes in hub modules. **a** A scatter plot of GS for EC versus the MM in the turquoise module. Intramodular analysis of the genes found in the turquoise module, which contains genes that have a high correlation with EC, with *p* < 4.6e-75 and correlation = 0.66. **b** Dendrogram of consensus module eigengenes obtained by WGCNA on the consensus correlation. **c** Heatmap plot of the adjacencies of modules. Red represents high adjacency (positive correlation) and blue represents low adjacency (negative correlation)

Moreover, an intramodular analysis of GS and MM of the genes in the 5 modules was followed. As GS and MM illustrated a very meaningful correlation, this finding intimated that the 590 genes in the turquoise module tend to be remarkably correlated with tumor among the 5 modules (Fig. 5a).

Interestingly, some of these gene modules had similar expression profiles. To find out the connections and interactions among these 5 co-expressed modules, we analyzed the connectivity of eigengenes. A cluster analysis was completed (Fig. 5b, c). In general, 5 clusters were classified into two clusters, and each contained two branches.

Gene Ontology was performed on these modules in order to explore the potential biological pathway that was correlated to EC. In GO analysis, we could find that DEGs in green module were mostly enriched in chemokine activity, chemokine receptor binding, glycosaminoglycan binding, CXCR chemokine receptor binding, serine-type endopeptidase activity, serine-type peptidase activity, serine hydrolase activity, endopeptidase activity, cytokine activity and heparin binding (Additional file 1: Fig. S6A). The DEGs in turquoise module were significantly enriched in microtubule motor activity, microtubule binding, tubulin binding, transcription factor activity, core promoter proximal region sequence-specific DNA binding, core promoter proximal region DNA binding, transcriptional repressor activity, RNA polymerase II transcription regulatory region sequence-specific binding, transcriptional activator activity, RNA polymerase II core promoter proximal region sequence-specific binding and RNA polymerase II core promoter proximal region sequence-specific DNA binding (Additional file 1: Fig. S6B). The DEGs in blue module were significantly enriched in cell adhesion molecule binding and cadherin binding (Additional file 1: Fig. S6C). The DEGs in brown module were significantly enriched in glycosaminoglycan binding and sulfur compound binding (Additional file 1: Fig. S6D). The DEGs in yellow module were significantly enriched in structural constituent of ribosome (Additional file 1: Fig. S6E). In KEGG analysis, we could find that DEGs in green module were mostly enriched in Leukocyte transendothelial migration, Chemokine signaling pathway, Leishmaniasis, Cytokine-cytokine receptor interaction, ECM-receptor interaction, Fluid shear stress and atherosclerosis and Focal adhesion (Additional file 1: Fig. S7A). The DEGs in turquoise module were significantly enriched in Cell cycle, Oocyte meiosis, Progesterone-mediated oocyte maturation, Cellular senescence, Fanconi anemia pathway and Human T-cell leukemia virus 1 infection (Additional file 1: Fig. S7B). The DEGs in blue module were significantly enriched in Glutathione metabolism (Additional file 1: Fig. S7C). The DEGs in brown module were significantly enriched in Cysteine and methionine metabolism and Pantothenate and CoA biosynthesis (Additional file 1: Fig. S7D). The DEGs in yellow module were significantly enriched in Ribosome and Bladder cancer (Additional file 1: Fig. S7E).

### Identification of hub genes in the turquoise module

**Fig. 6:**
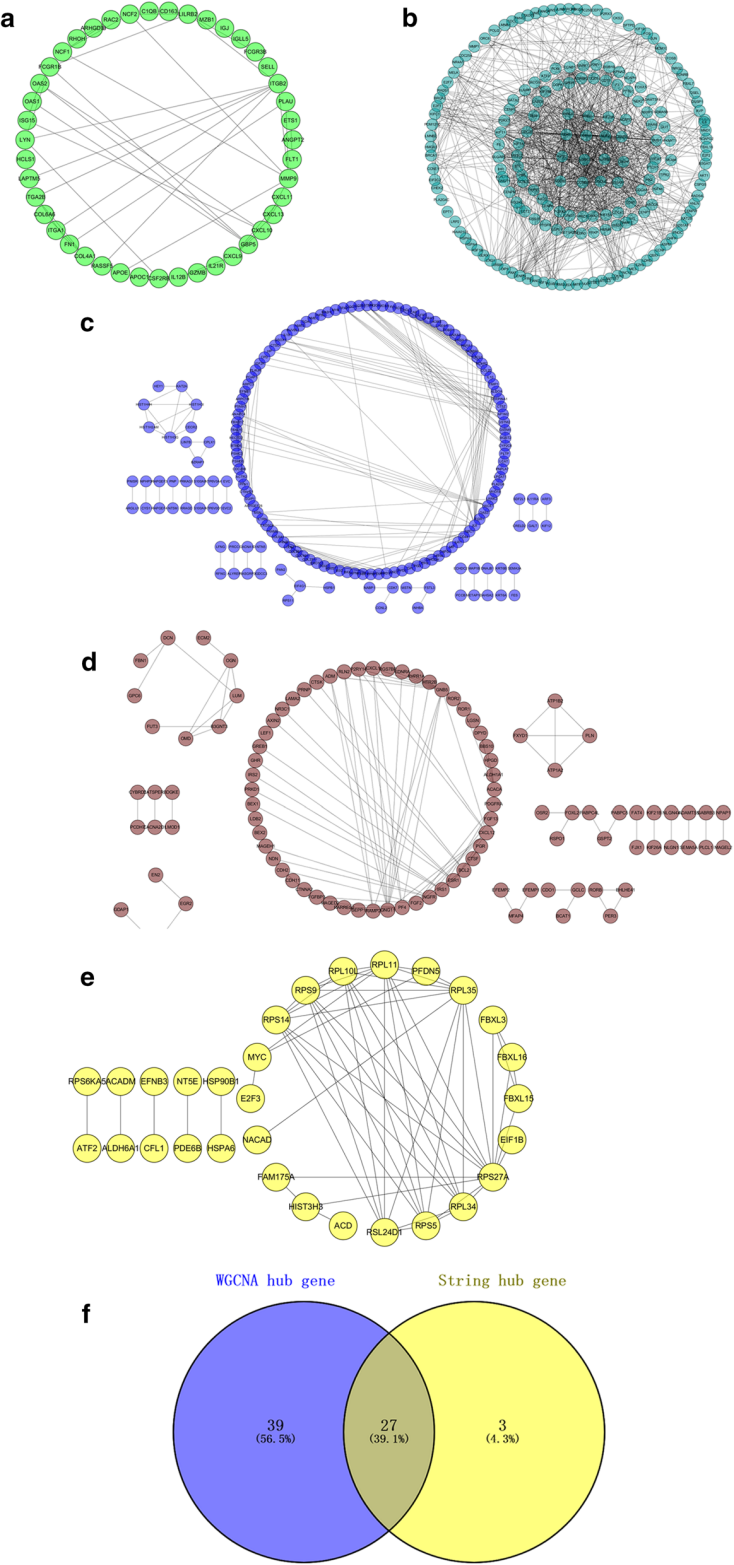
Comprehensive analysis of PPI and WGCNA hub modules. **a** PPI network of the green module. **b** PPI network of the turquoise module. **c** PPI network of the blue module. **d** PPI network of the brown module. **e** PPI network of the yellow module. **f** Real key genes belonging to both the turquoise module and the PPI network

According to the STRING database, we constructed a network of protein–protein interaction (PPI) for all genes in the 5 modules by Cytoscape respectively. The green module consisted of 87 nodes and 69 edges (Fig. 6a). The turquoise module consisted of 469 nodes and 1022 edges (Fig. 6b). The blue module consisted of 396 nodes and 273 edges (Fig. 6c). The brown module consisted of 289 nodes and 126 edges (Fig. 6d). The yellow module consisted of 120 nodes and 57 edges (Fig. 6e). Highly connected hub genes in a module played an important role in the biological processes. Defined by module connectivity, measured by absolute value of the Pearson's correlation (cor.geneModuleMembership > 0.8) and defined by cancer trait relationship, measured by absolute value of the Pearson's correlation (cor.geneTraitSignificance > 0.2), 65 genes with the high connectivity in 590 genes of turquoise module were taken as hub genes. As to the PPI network, we selected the top 30 DEGs with the highest connectivity as hub genes. Eventually, 27 hub genes were identified both in PPI network and co-expression network. These 27 hub genes were been regarded as “real” hub genes (Fig. 6f).

### Hub gene validation

All of the 27 hub genes were validated in TCGA data. We unearthed 11 prognostic genes and data showed that they all had higher expression in tumor tissues (*p* < 0.001) (Additional file 1: Fig. S8A–K). Survival analysis also showed that these 11 genes were negatively correlated with patient outcomes (Additional file 1: Fig. S9A–K).

To further demonstrate that the above 11 hub genes were related to EC, we used the GEPIA website to verify these 11 genes. We found that these 11 genes were highly expressed in cancer tissues, but the expression levels in normal tissues were significantly lower (Additional file 1: Fig. S10A–K).

We also used UALCAN (https://ualcan.path.uab.edu/analysis.html) to verify 11 genes. The results showed that the expression of these 11 genes in each stage of EC tissues was higher than that of normal tissues and increased in advanced tumors (Additional file 1: Fig. S11A–K). We further analyzed 11 genes of histological subtypes and found that compared with normal tissues, these 11 genes were highly expressed in endometrioid adenocarcinoma, serous carcinoma, Mixed serous and endometrioid adenocarcinoma respectively (Additional file 1: Fig. S12A–K).

Immunohistochemistry staining obtained from The Human Protein Atlas database also demonstrated the deregulations of real hub genes expression (Additional file 1: Figs. S13, S14) while NUF2 was not included in the website. In addition, ROC curve analysis was implemented to evaluate the capacity of real hub genes to distinguish EC and normal tissues by using SPSS (Additional file 1: Fig. S15A–K). Immunohistochemistry staining obtained from The Human Protein Atlas database showed consistent conclusions.

### Gene set enrichment analysis (GSEA)

**Fig. 7:**
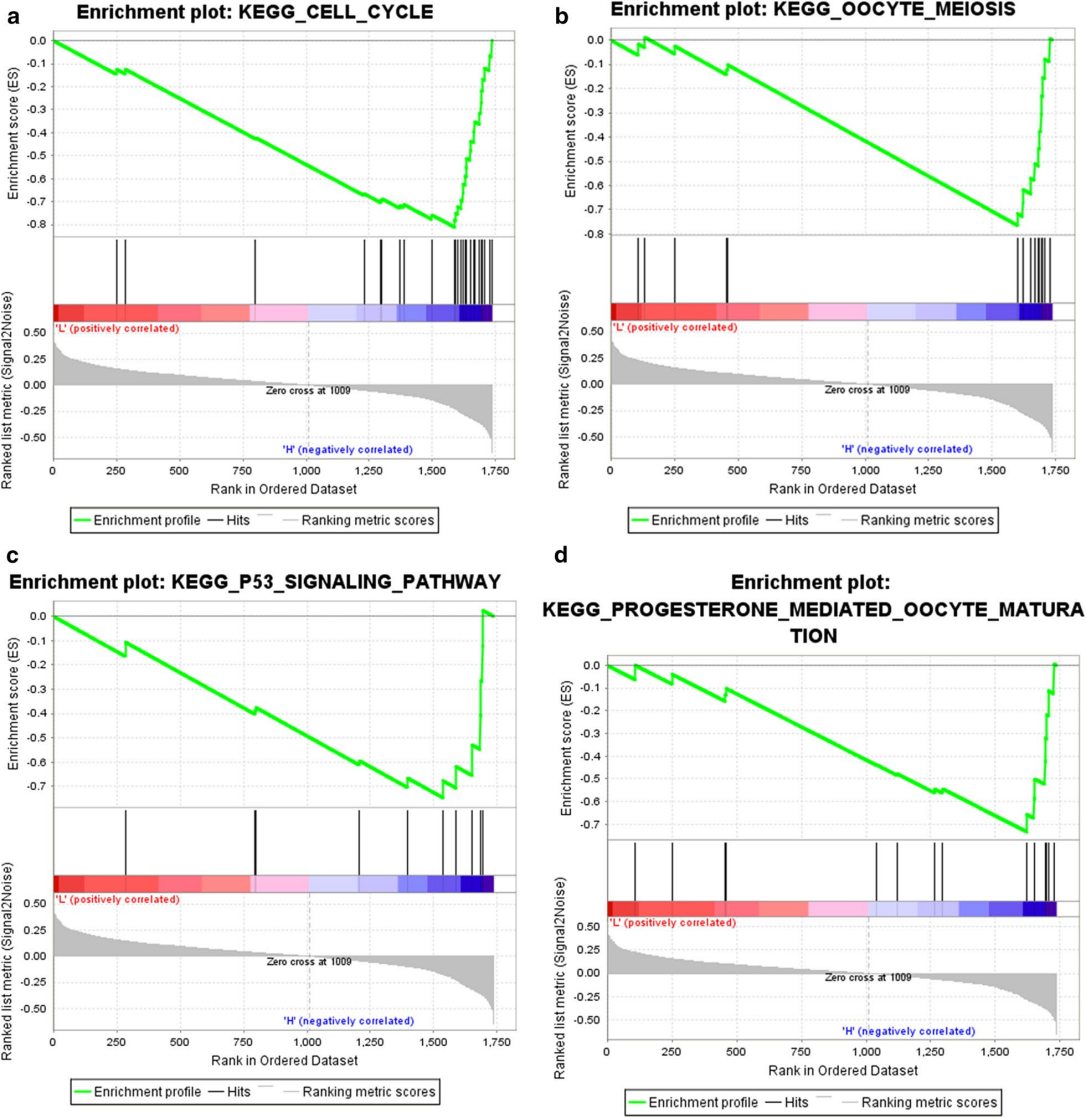
Gene set enrichment analysis (GSEA) using GSE17025. Only listed the four most common functional gene sets enriched in EC samples with hub genes highly expressed

To identify the potential function of the real hub genes in EC, GSEA was conducted to search KEGG pathways enriched in the highly-expressed samples. Four gene sets (n = 91), “Cell cycle”, “Oocyte meiosis”, “P53 signal pathway” and “progesterone mediated oocyte maturation” were enriched (FDR < 0.05) (Fig. 7a–d).

### Hub gene mutation validation

**Fig. 8:**
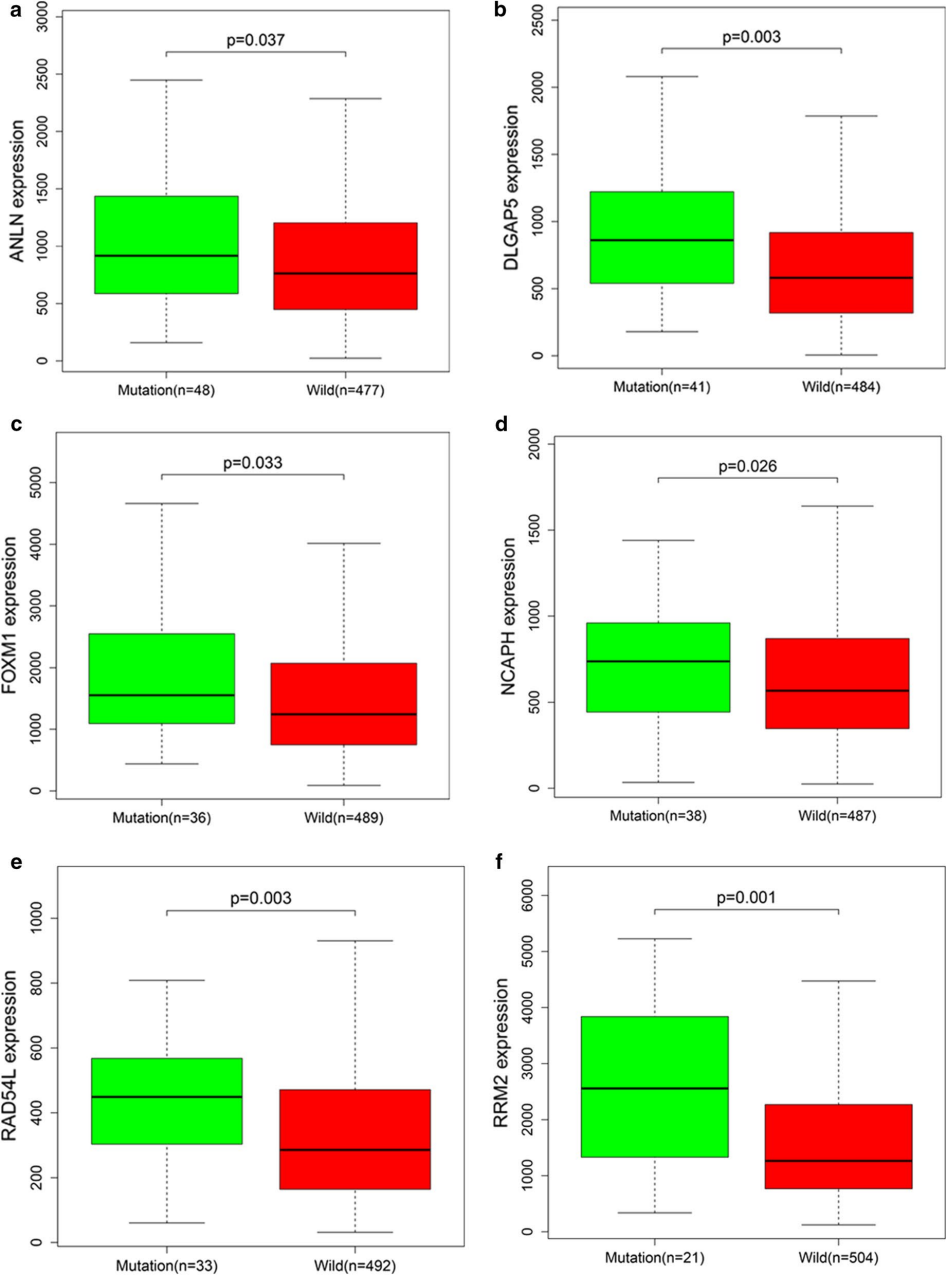
Hub genes mutation analysis. **a** ANLN. **b** DLGAP5. **c** FOXM1. **d** NCAPH. **e** RAD54L. **f** RRM2. They have significant mutations

**Fig. 9:**
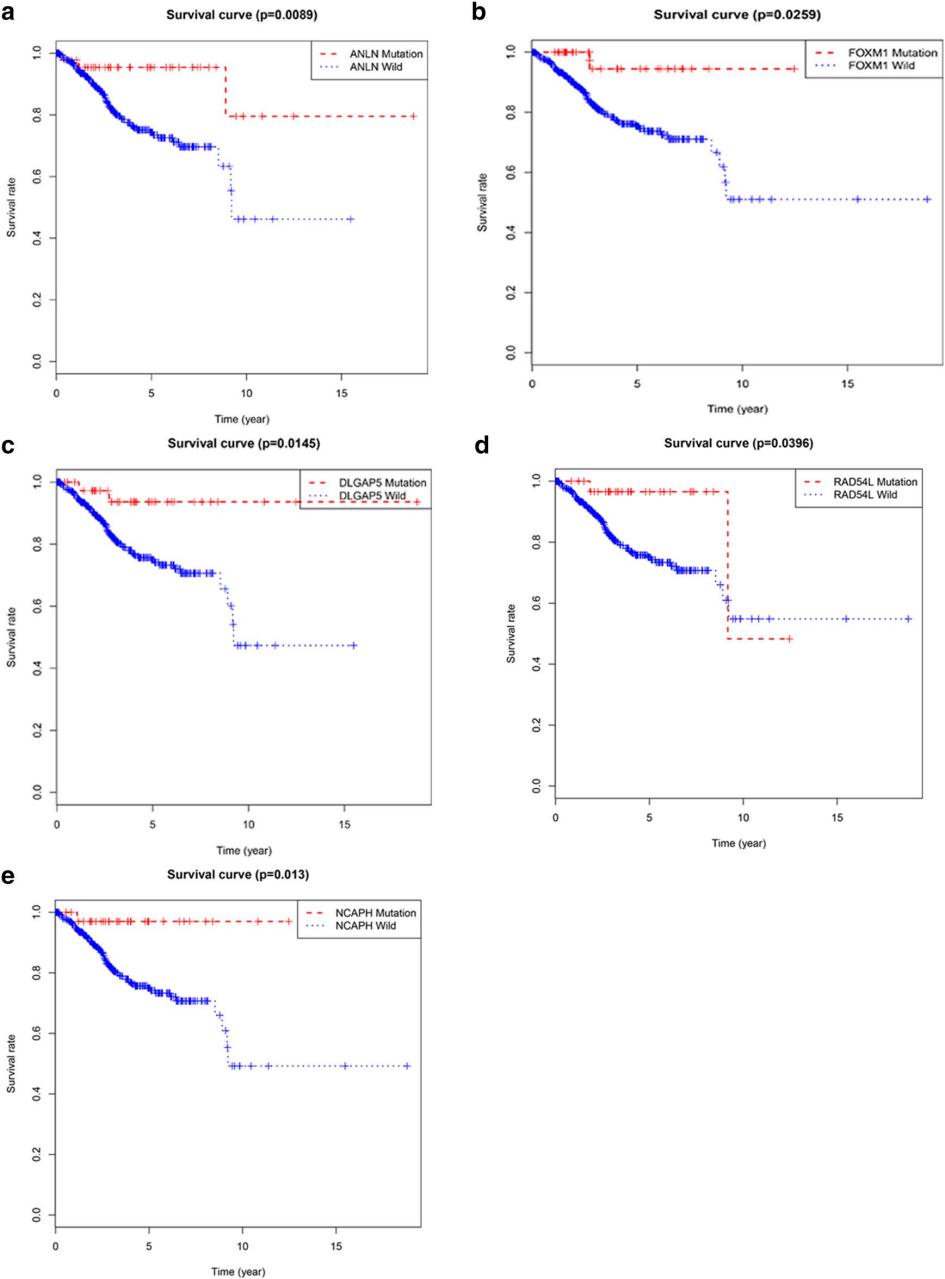
Survival analysis of mutated hub genes. **a** ANLN. **b** FOXM1. **c** DLGAP5. **d** RAD54L. **e** NCAPH. The overall survival of patients with genetic mutations is better than that of patients without genetic mutations

We used the TCGA dataset and the R language package to perform mutation analysis on the above 11 key genes. We found that ANLN, DLGAP5, FOXM1, NCAPH, RAD54L, and RRM2 had significant mutations (Fig. 8a–f), and ASPM, CDCA8, HJURP, HMMR, and NUF2 had no significant mutations. To further demonstrate the effect of gene mutations on EC, we performed survival analysis on the six mutant genes. Interestingly, for five of the six genes, the survival rate of patients with mutant genes was significantly higher than that of patients without gene mutations except RRM2 (Fig. 9a–e). This suggested that mutations in the above five genes may have a positive impact on the patient's prognosis.

## Discussion

Endometrial cancer is the most common malignancy of the female genital tract. Endometrial cancer predominately affects post-menopausal women, however 15–25% of cases are diagnosed before menopause. Endometrial cancer is not amenable to screening, hence needs to be managed effectively as soon as diagnosis is made. Therefore, it is very important to research biomarkers and related regulatory pathways that affect the development of EC. In this study, we examined the gene expression profile of GSE17025 including 91 tumor tissue samples and 12 normal tissue samples to identify the molecular mechanism of EC and seek some biomarkers. Bioinformatics analysis of these biological factors is used to seek genes that are beneficial to treatment.

In these findings, we identified 1737 DEGs associated with EC including 690 up-regulated genes and 1048 down-regulated genes using *p* < 0.05 and |logFC| ≥ 1 as the cutoff criteria. We utilized PPI network analysis and WGCNA analysis to select PPI and gene co-expression modules that were linked to the clinical development of EC. Furthermore, we conducted functional and pathway analysis to seek biological pathways that may have an impact on EC.

According to the GO analysis of DEGs, we found that upregulated DEGs were significantly enriched in CXCR chemokine receptor binding. The down-regulated genes were highly enriched in collagen binding. KEGG analysis showed that the upregulated genes were mostly enriched in IL-17 signaling pathway. The down-regulated genes were mostly enriched in MAPK signaling pathway. The PPI network was built on the DEGs that was analyzed by STRING website and Cytoscape software. Referring to cutoff of a k-core of 2, we found 8 clusters. Among the 8 clusters, cluster 1 got the highest score, included 32 nodes and 477 edges in this subnetwork.

WGCNA analysis showed that 11 modules possessed relevant expression pattern. For each module exceedingly, GO enrichment analysis was functioned to explore the biological process. The most relevant turquoise module contains 590 key genes. Through GO analysis, they were mainly enriched in microtubule motor activity. In KEGG analysis, we could find that DEGs in turquoise module were highly enriched in Cell cycle.

Sixty-five genes with the high connectivity in 590 genes of turquoise module were taken. Based on the PPI network, we screened 27 genes. Survival analysis of these 27 genes was performed by using the TCGA database, and 11 genes with significance were finally obtained. GEPIA website was used to verify these 11 genes. We found that these 11 genes were highly expressed in cancer tissues, but the expression levels of these genes in normal tissues were significantly lower than that in cancer tissues. We also used UALCAN to confirm the results, and we found that the expression levels of these 11 genes were higher in various subtypes of EC than in normal tissues. Interestingly, we further analyzed these 11 genes by using the TCGA database and the R language package and found that ANLN, DLGAP5, FOXM1, NCAPH, RAD54L, and RRM2 have mutational significance, and survival analysis showed that the survival rate of patients with the 5 mutant genes was higher than that of patients without gene mutations except RRM2. These results suggested that these five genes may influence the development of EC through the mechanism of mutation.

Anillin (ANLN), an actin-binding protein, was required for cytokinesis. Zhou W et al. found that knockdown of ANLN inhibits the growth and migration of human breast cancer cells [21]. Wang G et al. found overexpression of ANLN was correlated with colorectal cancer progression and poor prognosis [22]. Xia L et al. found that ANLN functions as a key candidate gene in cervical cancer through bioinformatics analysis [2]. Zeng S et al. used Transcriptome sequencing and then found that ANLN was a promising prognostic biomarker in bladder urothelial carcinoma [23].

DLGAP5 was also a frequently studied gene. Chen et al. suggested that DLGAP5 may be involved in the regulation of ovarian cancer as a key target of NOTCH3 [24]. Liu R et al. found that DLGAP5 was related to estrogen receptor positive breast cancer through WGCNA [25]. Liao et al. indicated that Silencing of DLGAP5 significant inhibits the proliferation and invasion of hepatocellular carcinoma cells [26]. Stangeland et al. found that DLGAP5 may be a biological target of glioblastoma stem cells through bioinformatics analysis [27]. Schneideret al. demonstrated that DLGAP5 was a specific mitosis-associated genes correlate with poor prognosis for non-small cell lung cancer patients [28].

The transcription factor Forkhead box protein M1 (FOXM1) was a preferred anticancer target, due to its significance in execution of mitosis, cell cycle progression, as well as other signal pathways leading to tumorigenesis [29]. Cui et al. demonstrated that FOXM1 promotes the Warburg effect and pancreatic cancer progression via transactivation of LDHA expression [30].

Ribonucleotide reductase subunit M2 (RRM2) has been shown to be a meaningful advance factor for advanced non-small cell lung cancer and breast cancer [31, 32]. Grolmusz et al. also found that Cell cycle dependent RRM2 may serve as proliferation marker and pharmaceutical target in adrenocortical cancer [33]. Wang et al. found that Increased expression of RRM2 by human papillomavirus E7 oncoprotein promotes angiogenesis in cervical cancer [34]. For gastric cancer cell, overexpression of RRM2 promotes their invasiveness via AKT/NF-κB signaling pathway [35]. NCAPH was proved highly expressed in colorectal cancer cell lines comparing with normal human colonic epithelial cells, and many NCAPH mutations in colorectal cancer patients were identified [36], but it was not detailed in EC. Similarly, RAD54L had also been shown to mutate in multiple tumors [37, 38].

ASPM proved to be positively correlated with the progress and poor prognosis of both prostate cancer and hepatocellular carcinoma [39, 40]. Wang et al. showed that ASPM promoted aggressiveness of pancreatic tumor [41]. ASPM has also been proven to affect the prognosis of patients with ovarian cancer in many ways [42, 43]. CDCA8 was often studied in bladder cancer and breast cancer, CDCA8 and FOXM1 appeared to be related in breast cancer [44, 45]. The specific mechanism of CDCA8 in EC was still unclear.

Holliday Junction Recognition Protein (HJURP) was a centromeric histone chaperone involved in de novo histone H3 variant CenH3 (CENP-A) recruitment. Cao et al. demonstrated that Silencing of HJURP induced dysregulation of cell cycle and ROS metabolism in bladder cancer cells via PPARγ-SIRT1 feedback loop [46]. HJURP proved to be an independent prognostic factor in Serous Ovarian Carcinoma and breast cancer [47, 48]. Chen et al. proved that HJURP promoted hepatocellular carcinoma proliferation by destabilizing p21 via the MAPK/ERK1/2 and AKT/GSK3β signaling pathways [49]. After Hu et al. knocked down NuF2 by siRNA, the proliferation of pancreatic cancer cells was inhibited [50]. NUF2 was certified to participate in the tumorigenicity of colon cancer cells [51]. Fu HL et al. found that silencing NUF2 inhibited the growth of osteosarcoma cells and promoted its apoptosis [52]. Experiments in glioma cells and hepatocellular carcinoma cells had the same results [53, 54]. The effects of HMMR had been mentioned in breast cancer and glioblastoma [55, 56].

## Conclusion

Through a series of comprehensive analysis of bioinformatics, we could roughly screen the hub genes and pathways related to the progression of EC, and target therapy for the extractive hub genes including ANLN, DLGAP5, FOXM1, NCAPH, RAD54L, RRM2, ASPM, CDCA8, HJURP, HMMR and NUF2. They might greatly promote the prognosis of EC. However, these key genes and pathways still need to be tested in a large quantity of clinical specimens, and need to be analyzed and validated in combination with the individual conditions of clinical patients in order to finally determine the biological targets that were most beneficial to endometrial cancer.

## Additional file

**Additional file 1: Fig. S1.** Identification of DEGs in EC and normal tissues. (A) The volcano plot of all DEGs. (B) Heatmap of the top 200 DEGs according to the value of |logFC|. **Fig. S2.** GO analysis and KEGG analysis of the DEGs. (A) In GO analysis, upregulated DEGs with fold change > 1. (B) In GO analysis, down-regulated DEGs with fold change > 1. (C) In KEGG analysis, upregulated DEGs with fold change > 1. (D) In KEGG analysis, down-regulated DEGs with fold change > 1. **Fig.S3.** GO analysis of the hub module. (A) Module rank 1. (B) Specific information of Module rank 1 (C) Module rank 2. (D) Specific information of Module rank 2. **Fig.S4.** KEGG analysis of the hub module. (A) Module rank 1. (B) Specific information of Module rank 1 (C) Module rank 2. (D) Specific information of Module rank 2. **Fig.S5.** Determination of soft-thresholding power in WGCNA. (A) Analysis of the scale-free fit index for various soft-thresholding powers (β). (B) Analysis of the mean connectivity for various soft-thresholding powers. (C) Histogram of connectivity distribution when β = 4. (D) Checking the scale free topology when β = 4. **Fig.S6.** GO enrichment analysis of 5 hub modules. (A) Green module. (B) Turquoise module. (C) Blue module. (D) Brown module. (E) Yellow module. **Fig.S7.** KEGG enrichment analysis of 5 hub modules. (A) Green module. (B) Turquoise module. (C) Blue module. (D) Brown module. (E) Yellow module. **Fig.S8.** Validation of 11 hub genes in TCGA databases. These 11 genes are all highly expressed in EC tissue samples compared with normal tissue samples. **Fig.S9.** Survival analysis of 11 hub genes. These 11 genes are all negative prognosis factors in EC, while patients with higher expression have significantly shorter overall survival. **Fig.S10.** Validation of 11 hub genes expression in GEPIA. Higher expression in tumor tissues compared with normal tissues. **Fig.S11.** Validation of 11 hub genes expression in UALCAN. The expression of 11 hub genes in EC tissues at different stages are all higher than normal tissues. (A) ANLN. (B)ASPM. (C) CDCA8. (D) DLGAP5. (E) FOXM1. (F) HJURP. (G) HMMR. (H) NCAPH. (I) NUF2. (J) RAD54L. (K) RRM2. **Fig.S12.** Validation of 11 hub genes expression in UALCAN. The expression of 11 hub genes in EC tissues of diffrent histological subtypes are all higher than normal tissues. (A) ANLN. (B)ASPM. (C) CDCA8. (D) DLGAP5. (E) FOXM1. (F) HJURP. (G) HMMR. (H) NCAPH. (I) NUF2. (J) RAD54L. (K) RRM2. **Fig.S13.** Immunohistochemistry of the five hub genes based on the Human Protein Atlas. (A) Protein levels of ANLN in tumor tissue (staining: High; intensity: Strong; quantity: 75–25%). Protein levels of ANLN in normal tissue (staining: Low; intensity: Moderate; quantity: < 25%; Location:Nuclear). (B) Protein levels of ASPM in tumor tissue (staining: Medium; intensity: Moderate; quantity: > 75%; Location: Cytoplasmic/membranou). Protein levels of ASPM in normal tissue (staining: Low; intensity: Weak; quantity: 75–25%; Location:Cytoplasmic/membranous). (C) Protein levels of CDCA8 in tumor tissue (staining: High; intensity: Strong; quantity: 75–25%; Location:Nuclear). Protein levels of CDCA8 in normal tissue (staining: Low; intensity: Moderate; quantity: Rare). (D) Protein levels of DLGAP5 in tumor tissue (staining: Medium; intensity: Strong; quantity: < 25%; Location: Cytoplasmic/membrano). Protein levels of DLGAP5 in normal tissue (staining: Low; intensity: Moderate; quantity: < 25%). (E) Protein levels of FOXM1 in tumor tissue (staining: high; intensity: Strong; quantity: > 75%; Location: Cytoplasmic/membranous nuclear). Protein levels of FOMX1 in normal tissue (staining: Medium; intensity: Moderate; quantity: > 75%). **Fig.S14.** Immunohistochemistry of the five hub genes based on the Human Protein Atlas. (A) Protein levels of HJURP in tumor tissue (staining: High; intensity: Moderate; quantity: 75–25%; Location:Cytoplasmic/membran). Protein levels of HJURP in normal tissue (staining: Low; intensity: moderate; quantity: < 25%; Location: Cytoplasmic/membranou) (B) Protein levels of HMMR in tumor tissue (staining: Low; intensity: moderate; quantity: < 25%). Protein levels of HMMR in normal tissue (staining: Not detected; intensity: Negative; quantity: Negative). (C) Protein levels of NCAPH in tumor tissue (staining: High; intensity: Strong; quantity: > 75%). Protein levels of NCAPH in normal tissue (staining: low; intensity: weak; quantity: 75–25%). (D) Protein levels of RAD54L in tumor tissue (staining: Medium; intensity: Moderate; quantity: > 75%; Location: Nuclear). Protein levels of RAD54L in normal tissue (staining: low; intensity: Moderate; quantity: < 25%). (E) Protein levels of RRM2 in tumor tissue (staining: Not detected; intensity: Not detected; quantity: < 25%). Protein levels of RRM2 in normal tissue (staining: Not detected; intensity: Negative; quantity: Negative). **Fig.S15.** Receiver operating characteristic (ROC) curve analysis and area under the curve (AUC) statistics was implemented to evaluate the capacity of real hub genes to distinguish EC and normal tissues. (A) ANLN. (B)ASPM. (C) CDCA8. (D) DLGAP5. (E) FOXM1. (F) HJURP. (G) HMMR. (H) NCAPH. (I) NUF2. (J) RAD54L. (K) RRM2.

## Data Availability

All data generated or analysed during this study are included in this published article.

## Abbreviations

EC: endometrial cancer
GEO: Gene Expression Omnibus
STRING: Search Tool for the Retrieval of Interacting Genes Database
PPI: protein–protein interaction
MCODE: Molecular Complex Detection
GSEA: gene set enrichment analysis
DEGs: differentially expressed genes
