# Supplementary material for: Eleven genes associated with progression and prognosis of endometrial cancer (EC) identified by comprehensive bioinformatics analysis
Source: Cancer Cell Int. 2019 May 20;19:136. doi: 10.1186/s12935-019-0859-1 (PMC6528324; doi:10.1186/s12935-019-0859-1)

**Fig.S1**. Identification of DEGs in EC and normal tissues. (A) The volcano plot of all DEGs. (B) Heatmap of the top 200 DEGs according to the value of |logFC|.

**Fig.S2**. GO analysis and KEGG analysis of the DEGs. (A) In GO analysis, upregulated DEGs with fold change > 1. (B) In GO analysis, down-regulated DEGs with fold change > 1. (C) In KEGG analysis, upregulated DEGs with fold change > 1. (D) In KEGG analysis, down-regulated DEGs with fold change > 1.

**Fig.S3**. GO analysis of the hub module. (A) Module rank 1. (B) Specific information of Module rank 1 (C) Module rank 2. (D) Specific information of Module rank 2.

**Fig.S4**. KEGG analysis of the hub module. (A) Module rank 1. (B) Specific information of Module rank 1 (C) Module rank 2. (D) Specific information of Module rank 2.

**Fig.S5**. Determination of soft-thresholding power in WGCNA. (A) Analysis of the scale-free fit index for various soft-thresholding powers (β). (B) Analysis of the mean connectivity for various soft-thresholding powers. (C) Histogram of connectivity distribution when β=4. (D) Checking the scale free topology when β=4.

**Fig.S6**. GO enrichment analysis of 5 hub modules. (A) Green module. (B) Turquoise module. (C) Blue module. (D) Brown module. (E) Yellow module.

**Fig.S7**. KEGG enrichment analysis of 5 hub modules. (A) Green module. (B) Turquoise module. (C) Blue module. (D) Brown module. (E) Yellow module.

**Fig.S8**. Validation of 11 hub genes in TCGA databases. These 11 genes are all highly expressed in EC tissue samples compared with normal tissue samples.

**Fig.S9**. Survival analysis of 11 hub genes. These 11 genes are all negative prognosis factors in EC, while patients with higher expression have significantly shorter overall survival.

**Fig.S10**. Validation of 11 hub genes expression in GEPIA. Higher expression in tumor tissues compared with normal tissues.

**Fig.S11**. Validation of 11 hub genes expression in UALCAN. The expression of 11 hub genes in EC tissues at different stages are all higher than normal tissues. (A) ANLN. (B)ASPM. (C) CDCA8. (D) DLGAP5. (E) FOXM1. (F) HJURP. (G) HMMR. (H) NCAPH. (I) NUF2. (J) RAD54L. (K) RRM2.

**Fig.S12**. Validation of 11 hub genes expression in UALCAN. The expression of 11 hub genes in EC tissues of diffrent histological subtypes are all higher than normal tissues. (A) ANLN. (B)ASPM. (C) CDCA8. (D) DLGAP5. (E) FOXM1. (F) HJURP. (G) HMMR. (H) NCAPH. (I) NUF2. (J) RAD54L. (K) RRM2.

**Fig.S13**. Immunohistochemistry of the five hub genes based on the Human Protein Atlas. (A) Protein levels of ANLN in tumor tissue (staining: High; intensity: Strong; quantity: 75%-25%). Protein levels of ANLN in normal tissue (staining: Low; intensity: Moderate; quantity: <25%; Location: Nuclear). (B) Protein levels of ASPM in tumor tissue (staining: Medium; intensity: Moderate; quantity: >75%; Location: Cytoplasmic/membranou). Protein levels of ASPM in normal tissue (staining: Low; intensity: Weak; quantity: 75%-25%; Location: Cytoplasmic/membranous). (C) Protein levels of CDCA8 in tumor tissue (staining: High; intensity: Strong; quantity: 75%-25%; Location: Nuclear). Protein levels of CDCA8 in normal tissue (staining: Low; intensity: Moderate; quantity: Rare). (D) Protein levels of DLGAP5 in tumor tissue (staining: Medium; intensity: Strong; quantity: <25%; Location: Cytoplasmic/membrano). Protein levels of DLGAP5 in normal tissue (staining: Low; intensity: Moderate; quantity: <25%). (E) Protein levels of FOXM1 in tumor tissue (staining: high; intensity: Strong; quantity: >75%; Location: Cytoplasmic/membranous nuclear). Protein levels of FOMX1 in normal tissue (staining: Medium; intensity: Moderate; quantity: >75%).

**Fig.S14**. Immunohistochemistry of the five hub genes based on the Human Protein Atlas. (A) Protein levels of HJURP in tumor tissue (staining: High; intensity: Moderate; quantity: 75%-25%; Location: Cytoplasmic/membran). Protein levels of HJURP in normal tissue (staining: Low; intensity: moderate; quantity: <25%; Location: Cytoplasmic/membranou) (B) Protein levels of HMMR in tumor tissue (staining: Low; intensity: moderate; quantity: <25%). Protein levels of HMMR in normal tissue (staining: Not detected; intensity: Negative; quantity: Negative). (C) Protein levels of NCAPH in tumor tissue (staining: High; intensity: Strong; quantity: > 75%). Protein levels of NCAPH in normal tissue (staining: low; intensity: weak; quantity: 75%-25%). (D) Protein levels of RAD54L in tumor tissue (staining: Medium; intensity: Moderate; quantity: > 75%; Location: Nuclear). Protein levels of RAD54L in normal tissue (staining: low; intensity: Moderate; quantity: <25%). (E) Protein levels of RRM2 in tumor tissue (staining: Not detected; intensity: Not detected; quantity: <25%). Protein levels of RRM2 in normal tissue (staining: Not detected; intensity: Negative; quantity: Negative).

**Fig.S15**. Receiver operating characteristic (ROC) curve analysis and area under the curve (AUC) statistics was implemented to evaluate the capacity of real hub genes to distinguish EC and normal tissues. (A) ANLN. (B)ASPM. (C) CDCA8. (D) DLGAP5. (E) FOXM1. (F) HJURP. (G) HMMR. (H) NCAPH. (I) NUF2. (J) RAD54L. (K) RRM2.


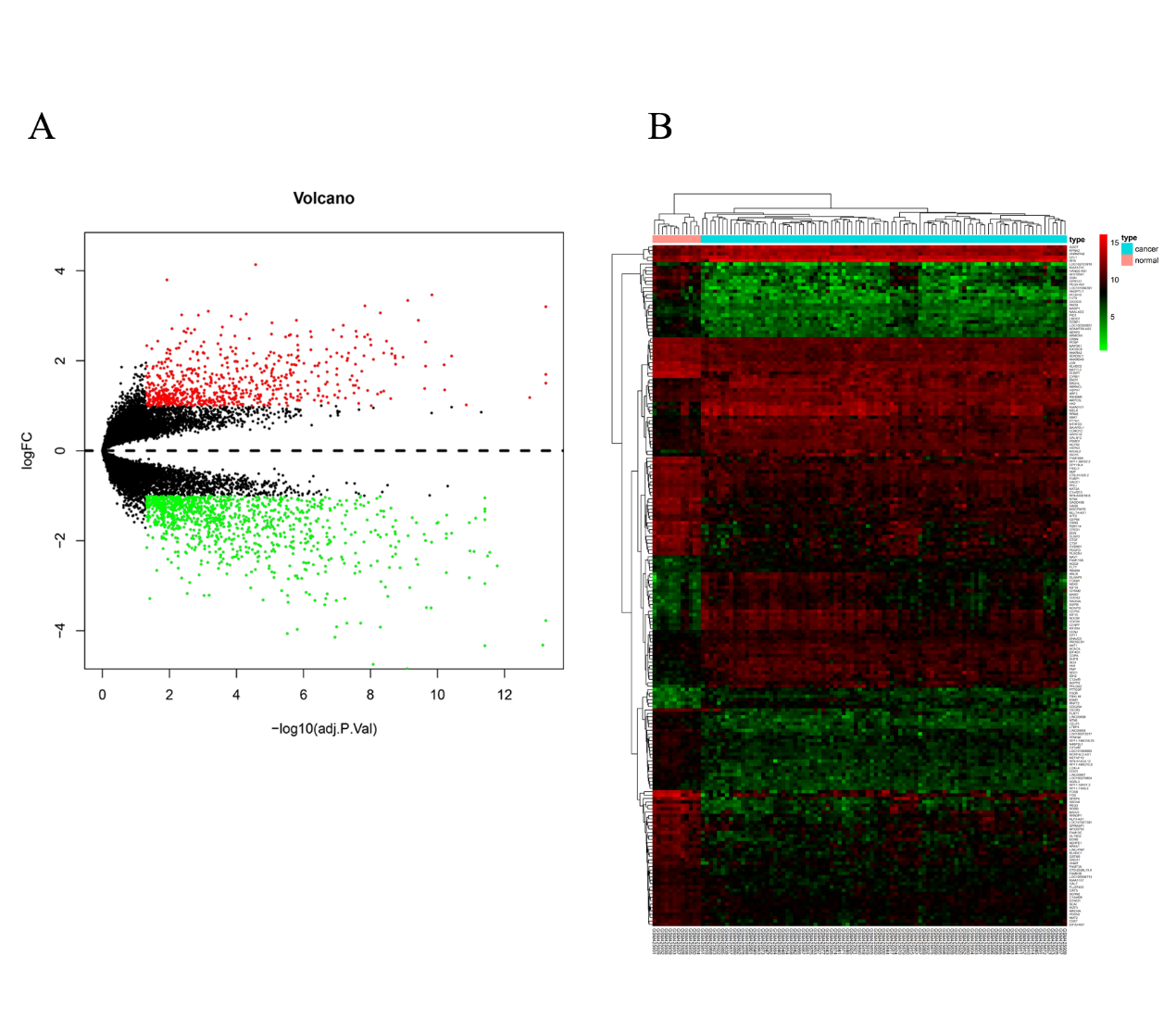

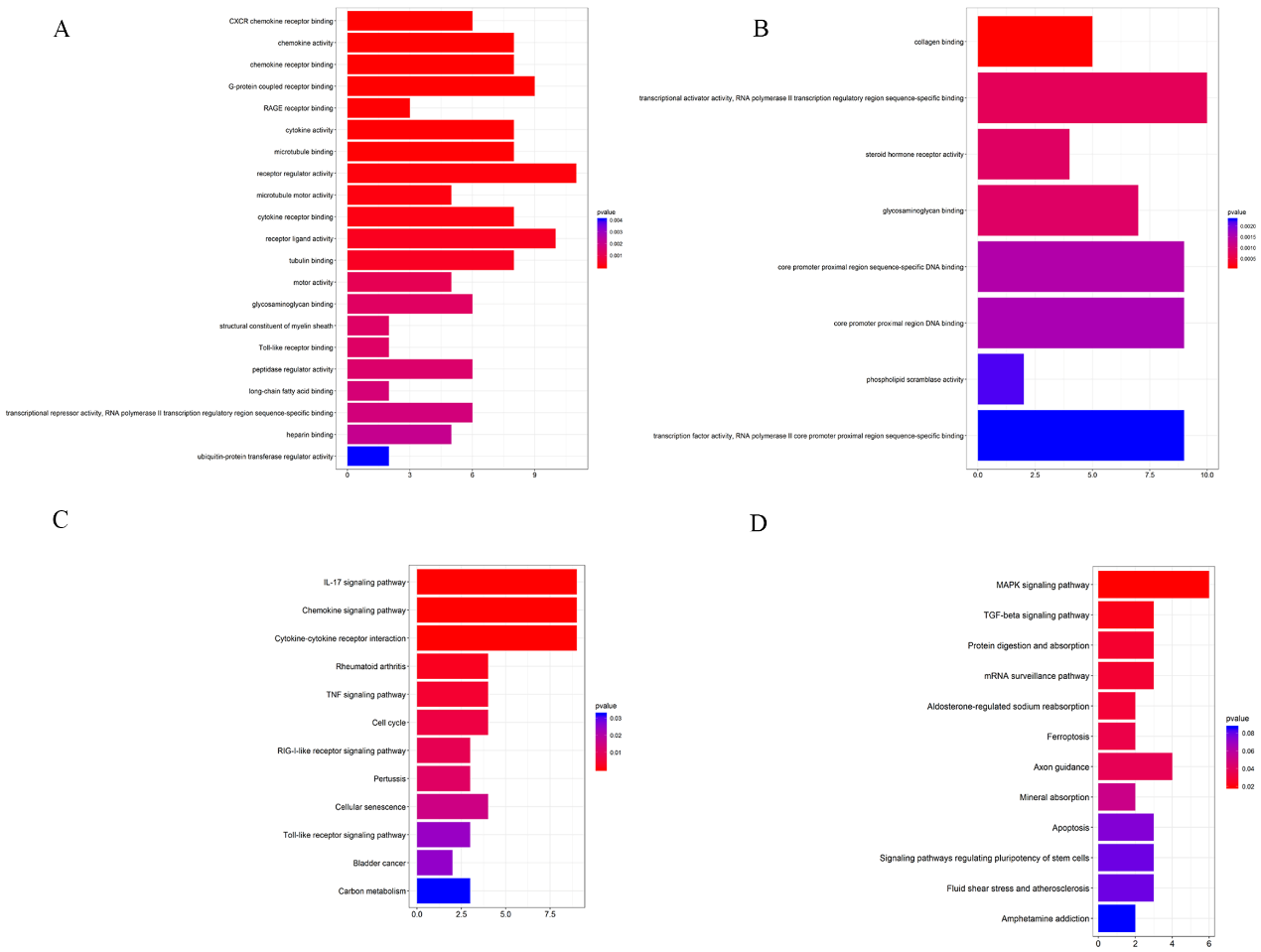

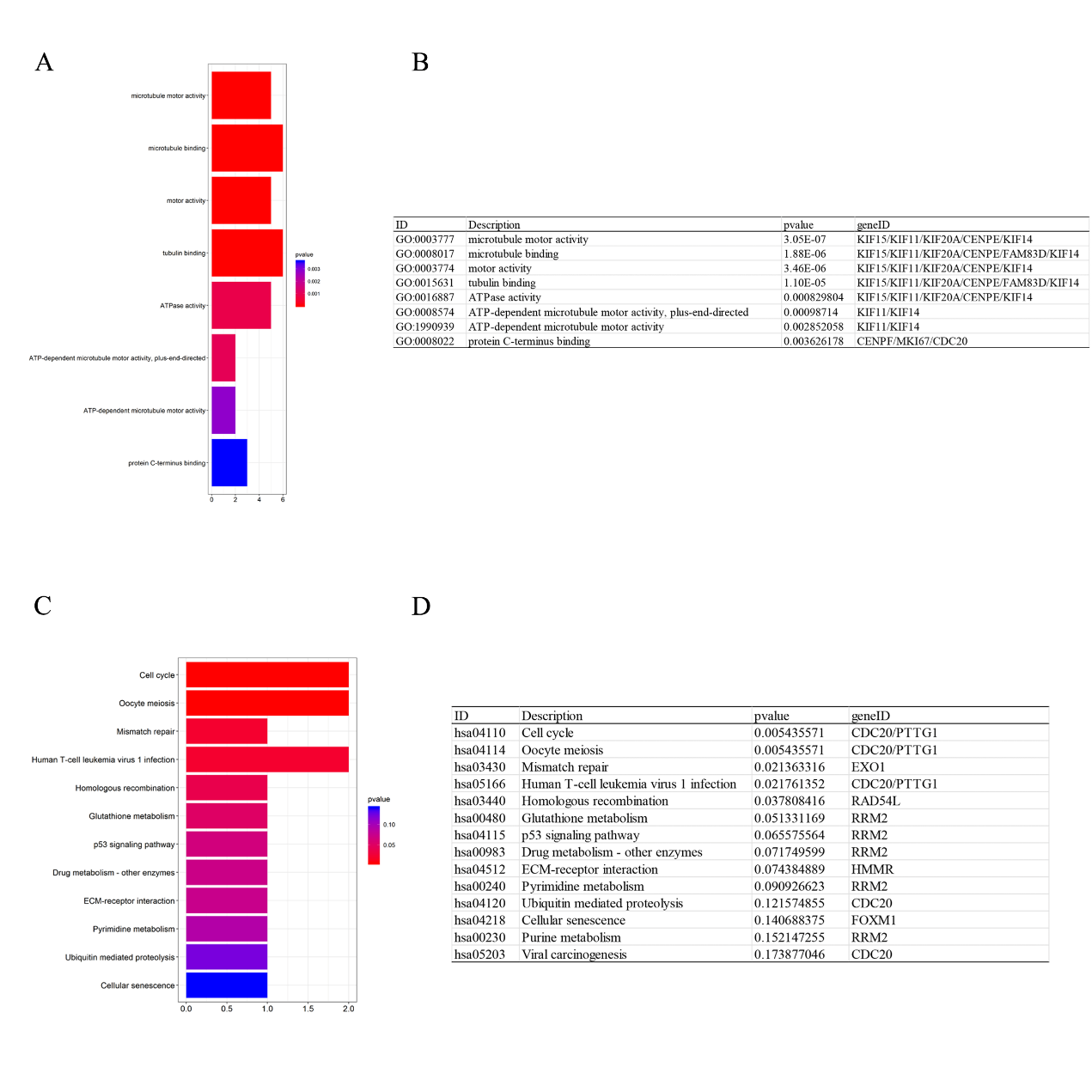

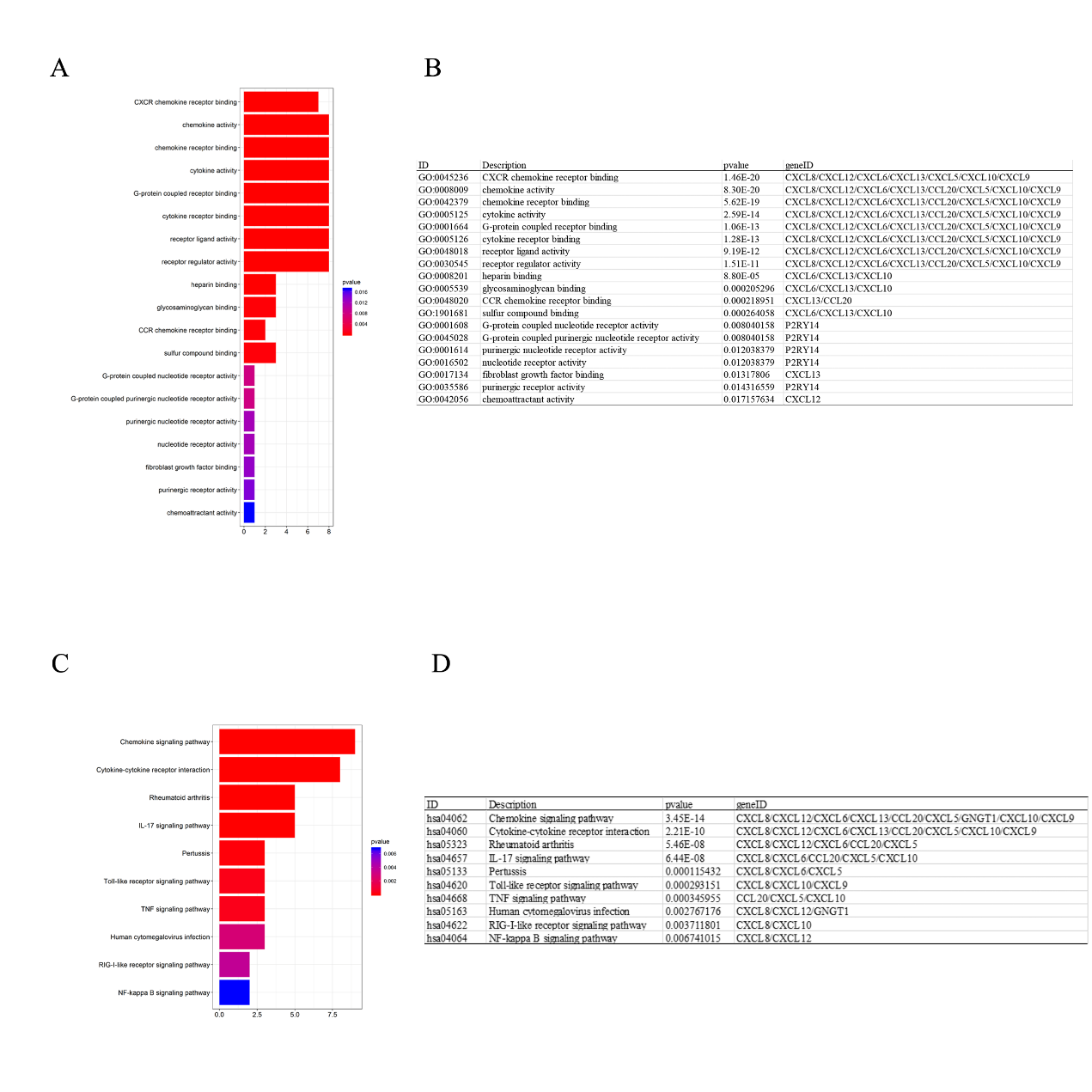

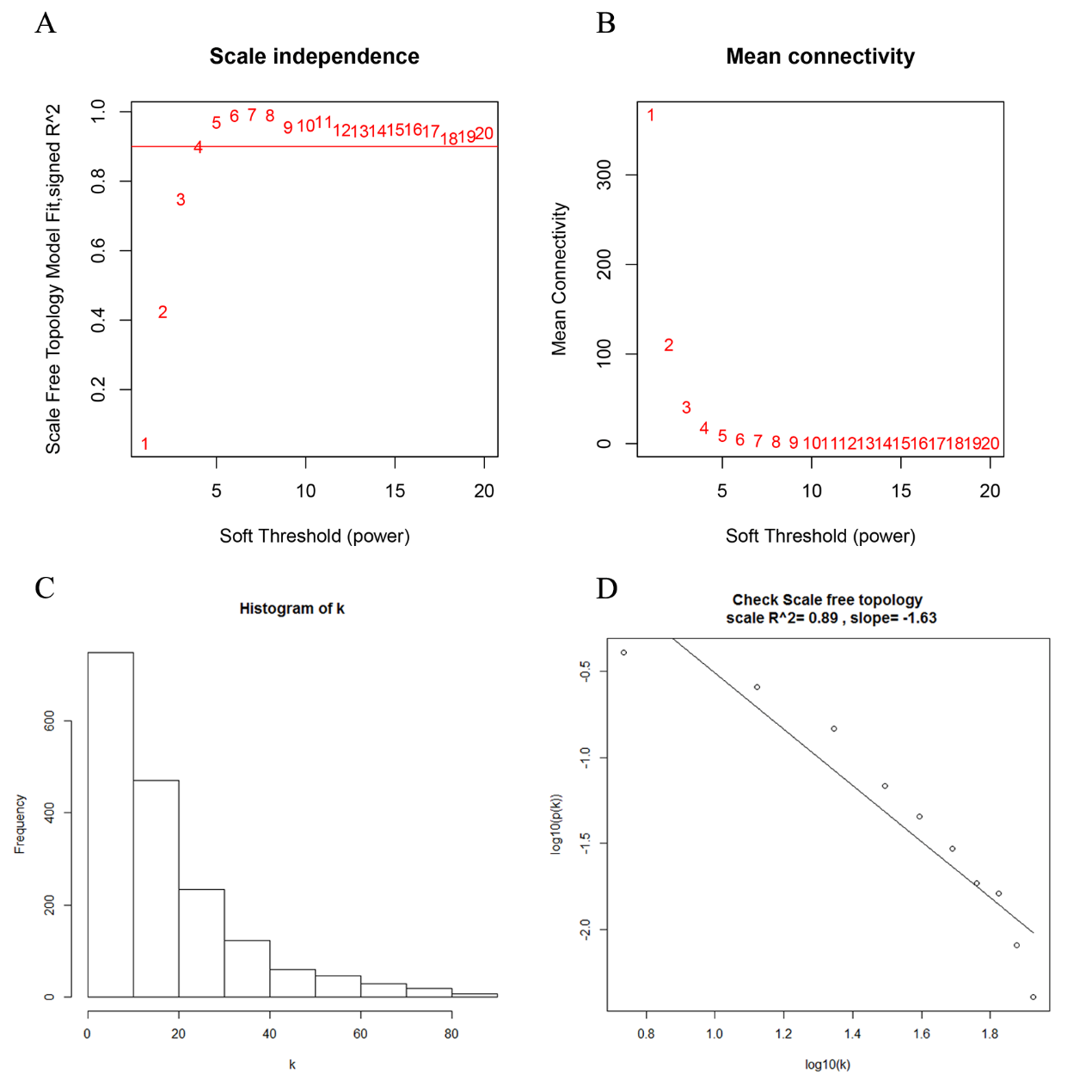

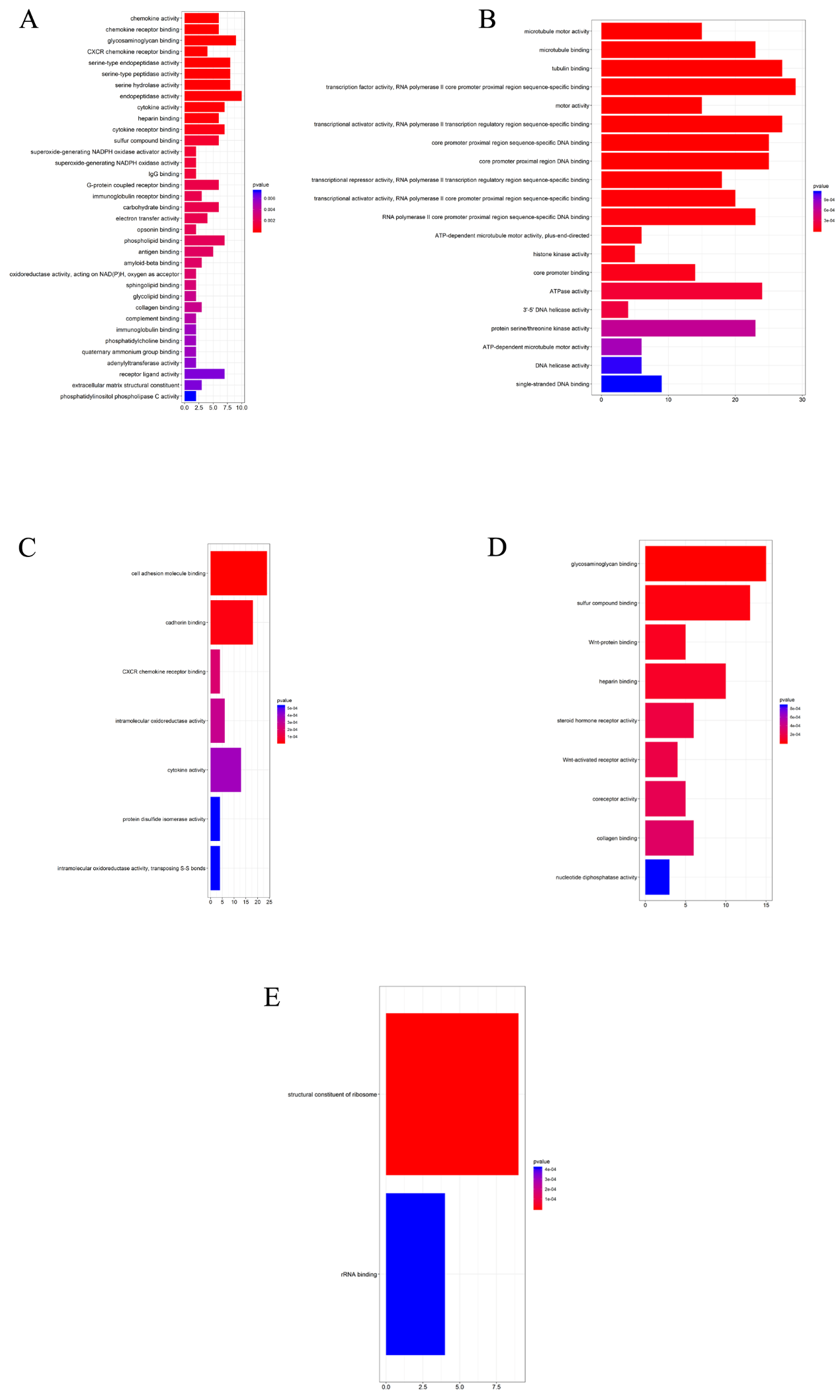

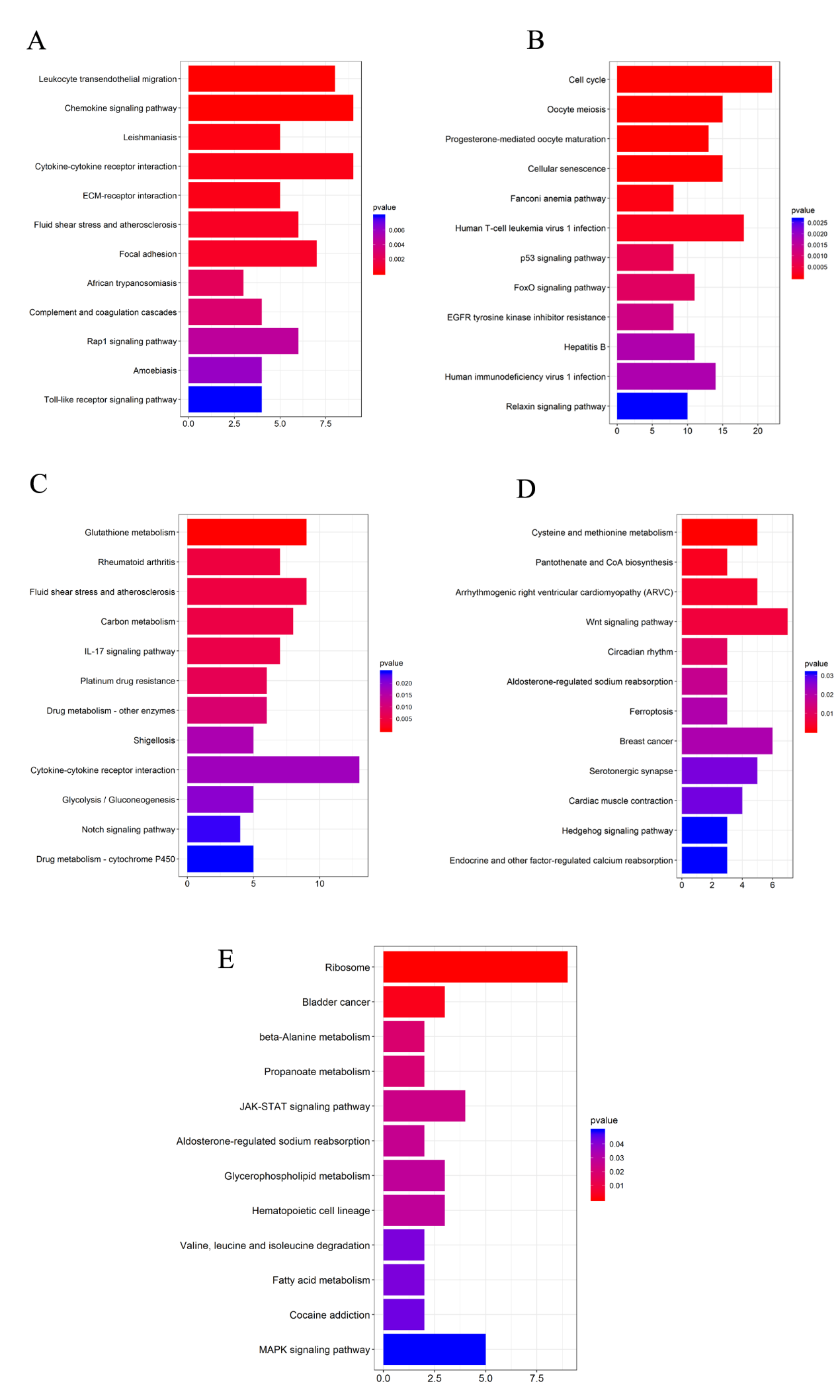

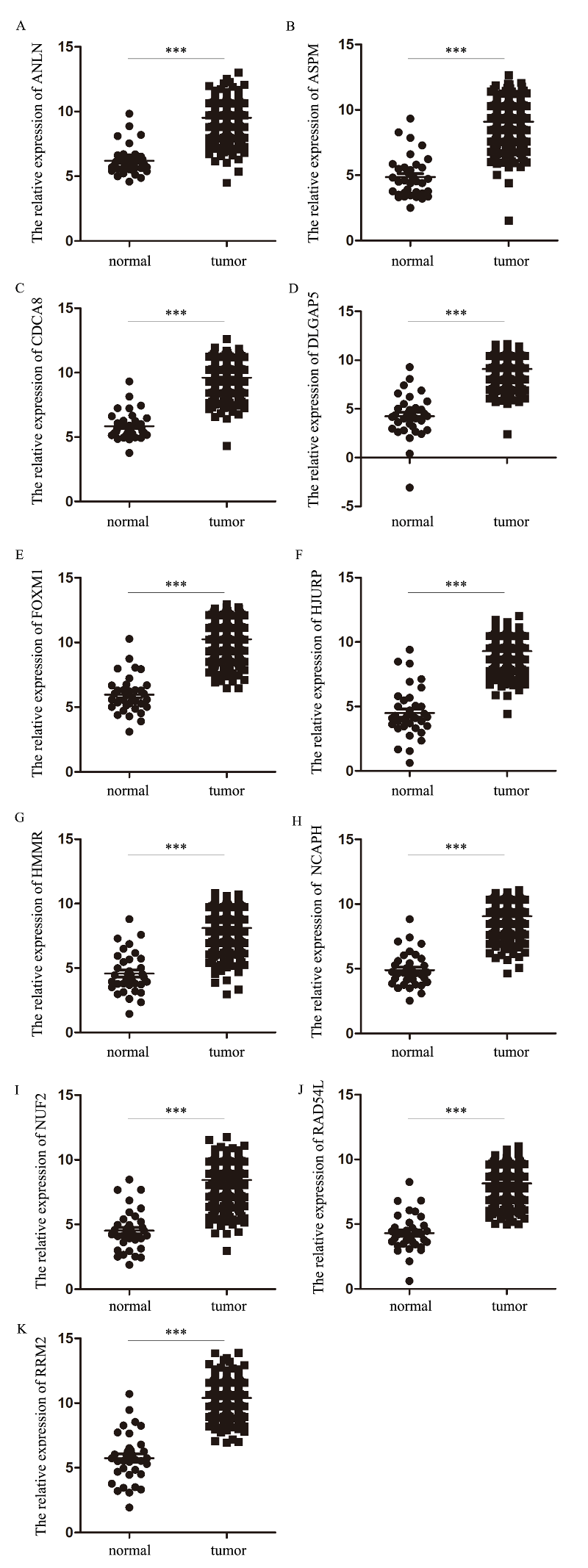

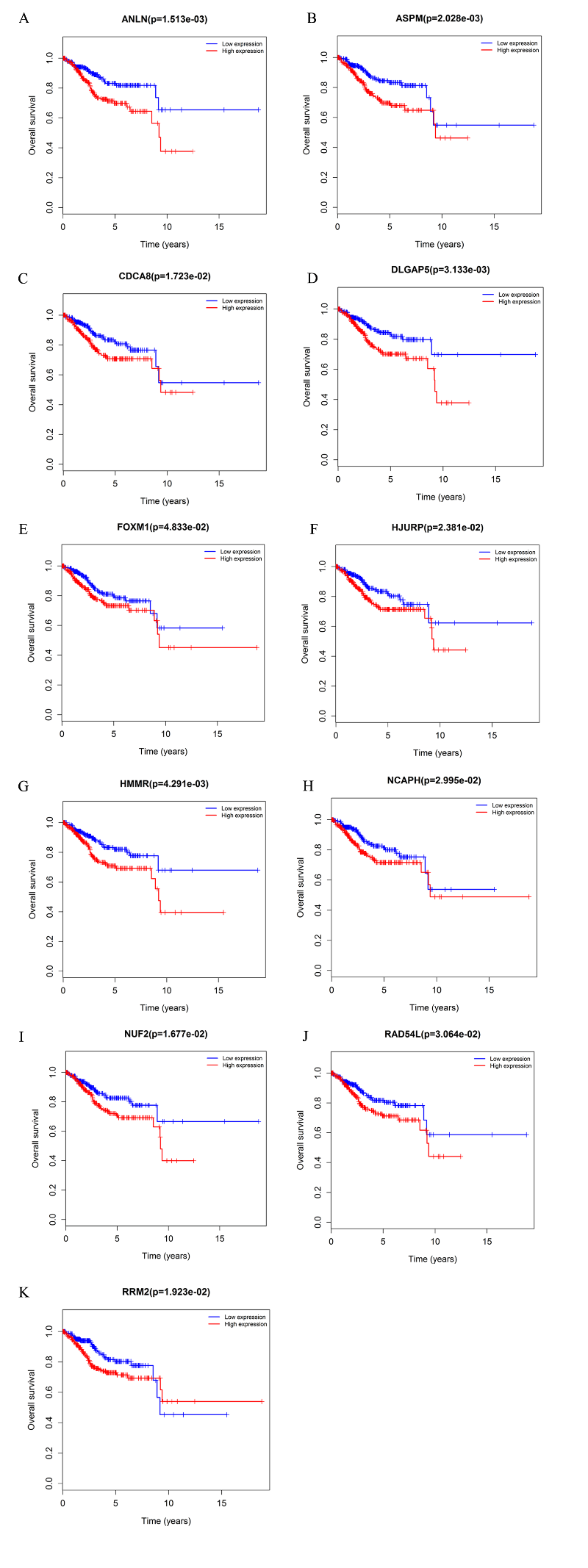

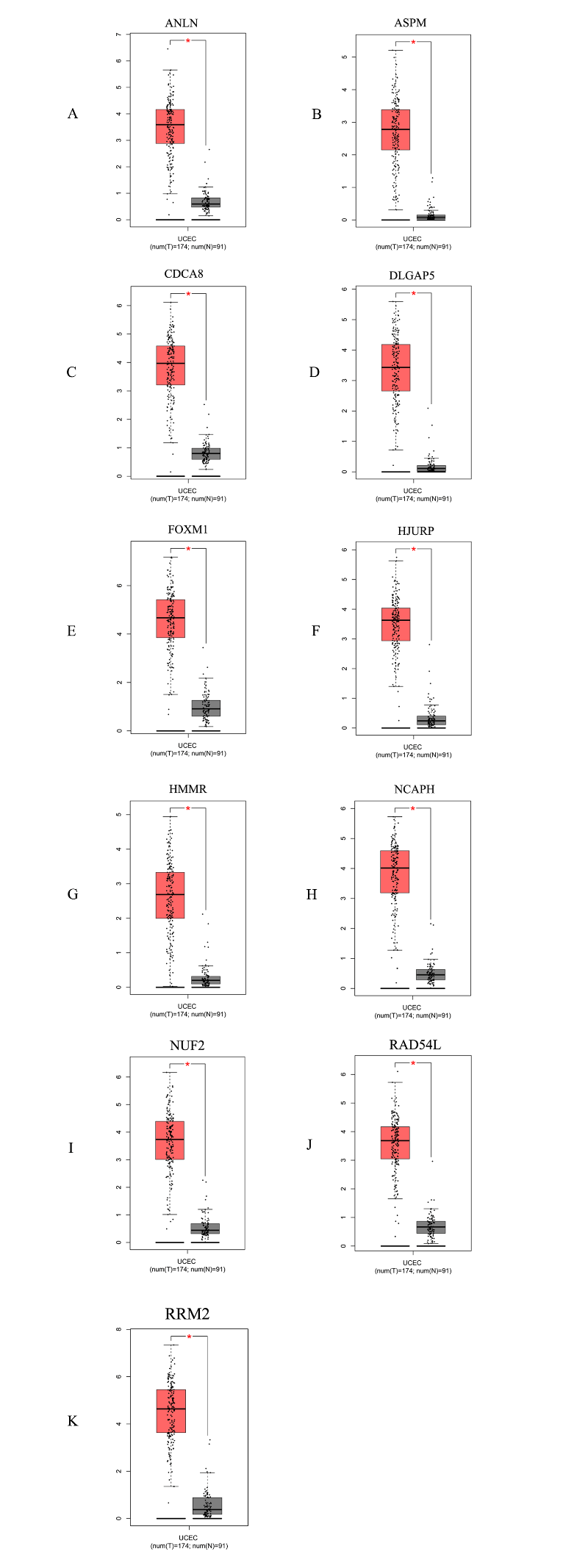

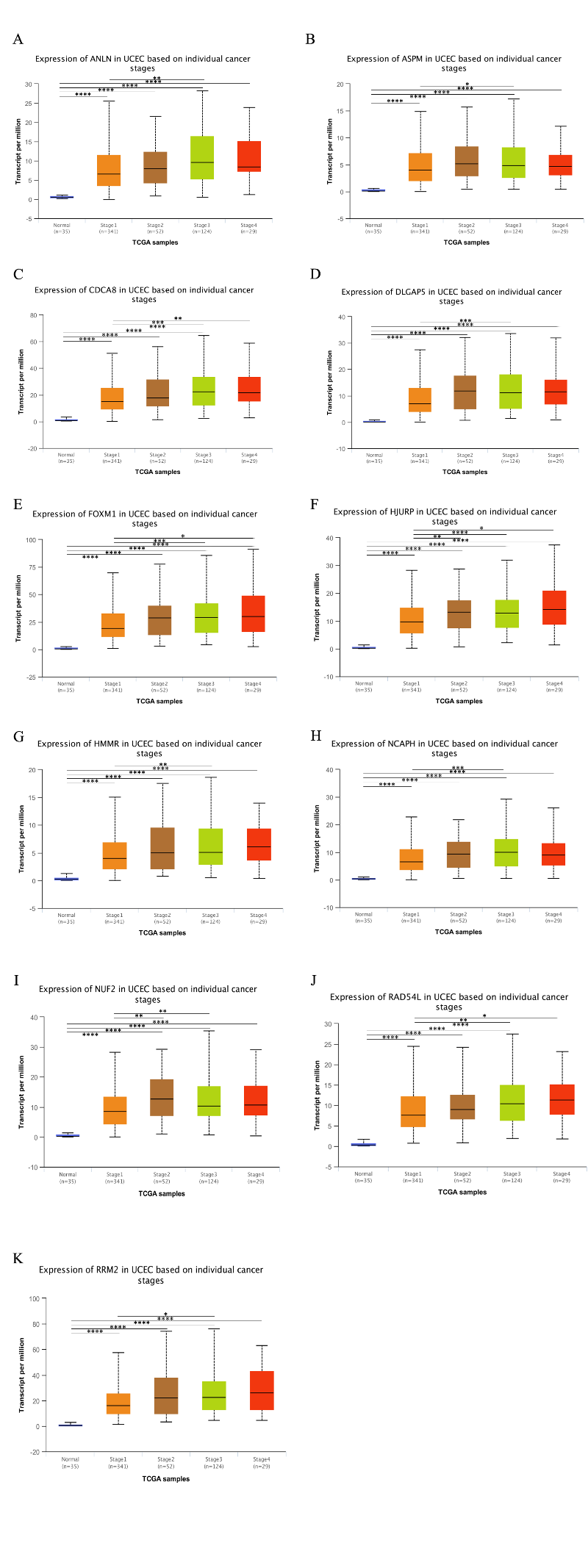

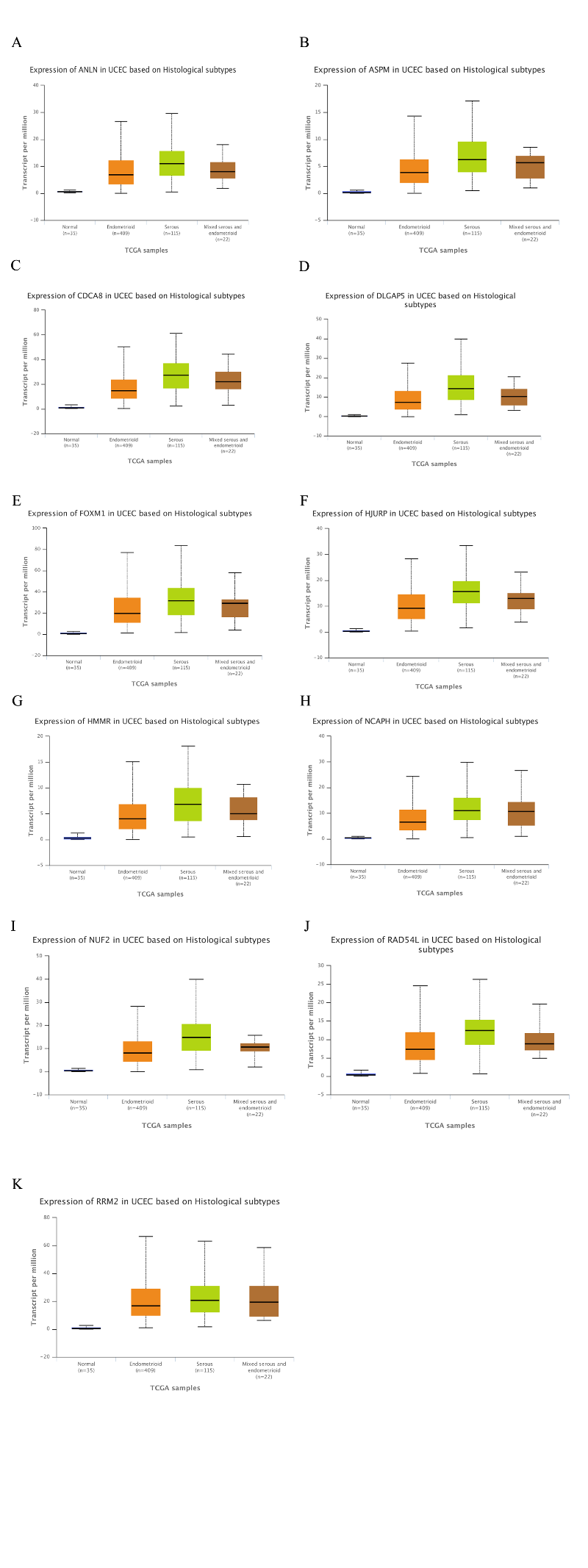

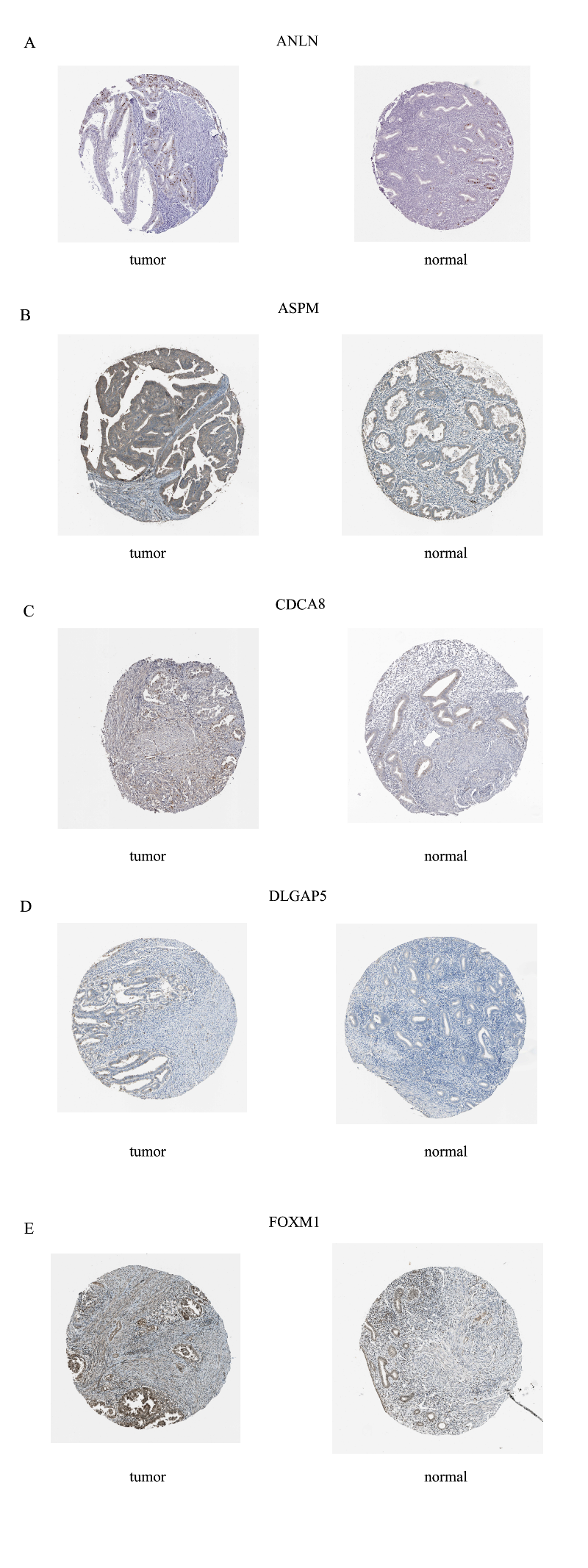

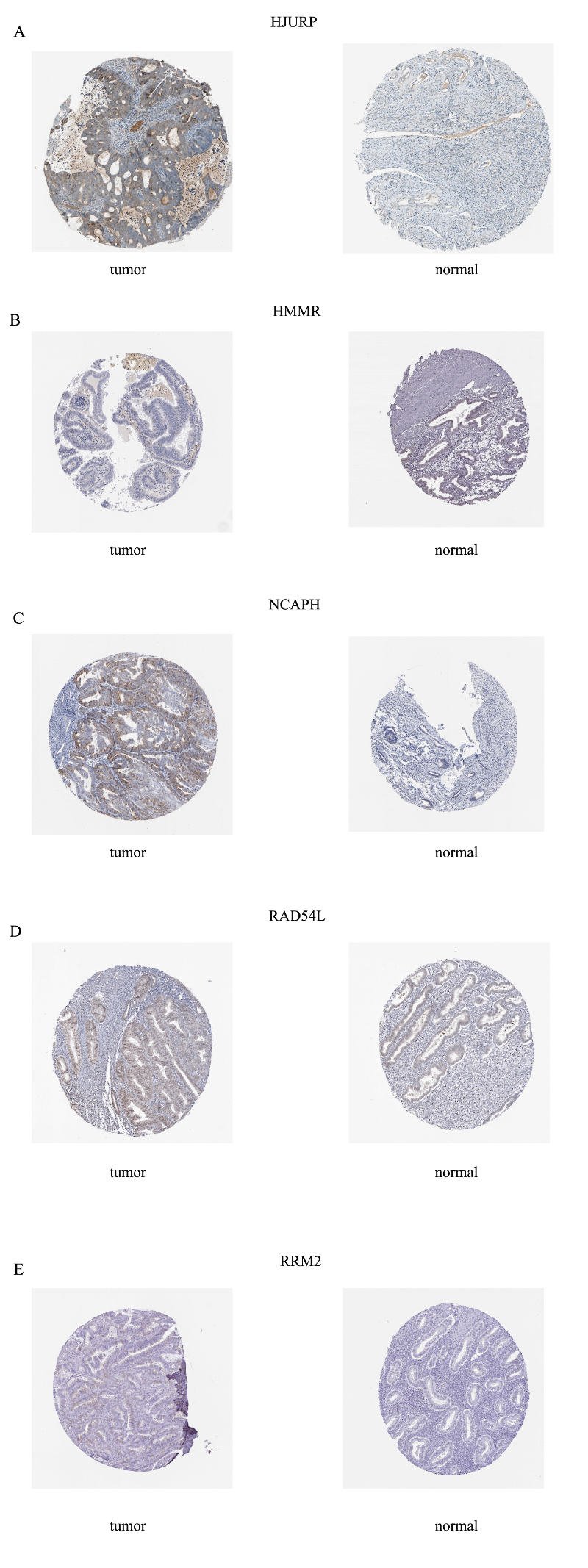

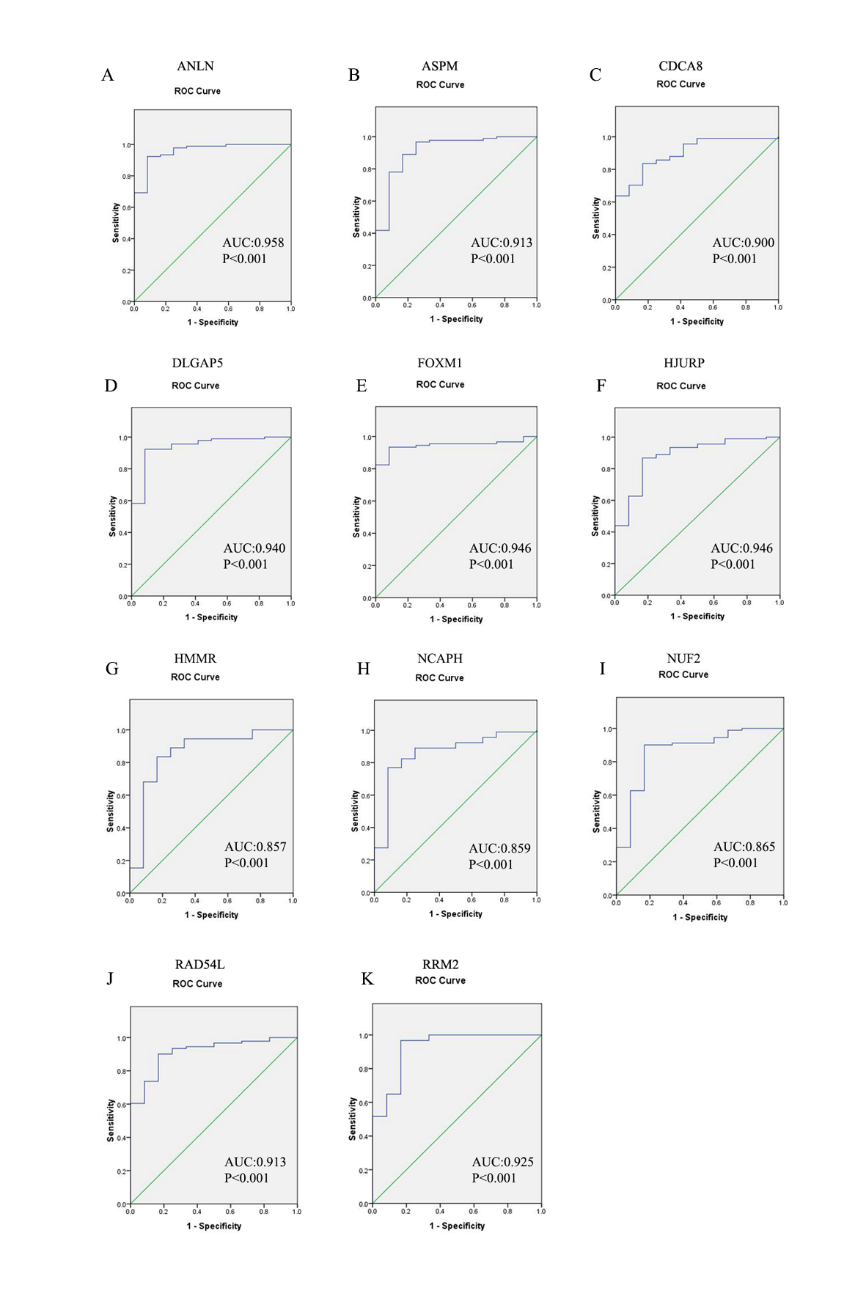

Supplement: Supplementary file 1 — Additional file 1: Fig. S1. Identification of DEGs in EC and normal tissues. (A) The volcano plot of all DEGs. (B) Heatmap of the top 200 DEGs according to the value of |logFC|. Fig. S2. GO analysis and KEGG analysis of the DEGs. (A) In GO analysis, upregulated DEGs with fold change > 1. (B) In GO analysis, down-regulated DEGs with fold change > 1. (C) In KEGG analysis, upregulated DEGs with fold change > 1. (D) In KEGG analysis, down-regulated DEGs with fold change > 1. Fig.S3. GO analysis of the hub module. (A) Module rank 1. (B) Specific information of Module rank 1 (C) Module rank 2. (D) Specific information of Module rank 2. Fig.S4. KEGG analysis of the hub module. (A) Module rank 1. (B) Specific information of Module rank 1 (C) Module rank 2. (D) Specific information of Module rank 2. Fig.S5. Determination of soft-thresholding power in WGCNA. (A) Analysis of the scale-free fit index for various soft-thresholding powers (β). (B) Analysis of the mean connectivity for various soft-thresholding powers. (C) Histogram of connectivity distribution when β = 4. (D) Checking the scale free topology when β = 4. Fig.S6. GO enrichment analysis of 5 hub modules. (A) Green module. (B) Turquoise module. (C) Blue module. (D) Brown module. (E) Yellow module. Fig.S7. KEGG enrichment analysis of 5 hub modules. (A) Green module. (B) Turquoise module. (C) Blue module. (D) Brown module. (E) Yellow module. Fig.S8. Validation of 11 hub genes in TCGA databases. These 11 genes are all highly expressed in EC tissue samples compared with normal tissue samples. Fig.S9. Survival analysis of 11 hub genes. These 11 genes are all negative prognosis factors in EC, while patients with higher expression have significantly shorter overall survival. Fig.S10. Validation of 11 hub genes expression in GEPIA. Higher expression in tumor tissues compared with normal tissues. Fig.S11. Validation of 11 hub genes expression in UALCAN. The expression of 11 hub genes in EC tissues at different stages are all [file 12935_2019_859_MOESM1_ESM.docx]
